# TGF‐β/Smad‐Associated Epithelial–Fibroblast Remodeling Underlies Collagen Deposition in Chronic Rhinosinusitis With Nasal Polyps

**DOI:** 10.1002/iid3.70532

**Published:** 2026-10-06

**Authors:** Shilong Liu, Peizheng Xie, Hanwei Deng, Zhenhua Zhu

**Affiliations:** ^1^ The First Clinical College of Traditional Chinese Medicine Hunan University of Traditional Chinese Medicine Changsha China; ^2^ Department of Otorhinolaryngology–Head and Neck Surgery First Affiliated Hospital of Hunan University of Traditional Chinese Medicine Changsha Hunan China

**Keywords:** dexamethasone, epithelial–mesenchymal transition, fibroblast activation, macrophage, nasal polyps, single‐cell RNA sequencing

## Abstract

**Background:**

Chronic rhinosinusitis with nasal polyps (CRSwNP) is frequently accompanied by epithelial barrier disruption and tissue remodeling. This study aimed to investigate whether epithelial–mesenchymal transition (EMT), fibroblast activation, collagen deposition, and TGF‐β/Smad signaling are coordinated during CRSwNP nasal mucosal remodeling.

**Materials and Methods:**

Publicly available single‐cell RNA sequencing datasets from two independent human cohorts were integrated to characterize epithelial, fibroblast, and myeloid heterogeneity, disease‐associated transcriptional changes, pseudotime dynamics, and cell–cell communication, with CellChat applied to the COLLAGEN and TGF‐β signaling modules. Immunofluorescence, Masson's trichrome, and periodic acid–Schiff (PAS) staining were performed on nasal polyps from CRSwNP patients and control nasal mucosa. An ovalbumin (OVA)/*Staphylococcus aureus* enterotoxin B (SEB)‐induced CRSwNP‐like mouse model, including a dexamethasone intervention arm, was used for histopathological, immunofluorescence, and Western blot validation.

**Results:**

In CRSwNP, fibroblasts shifted toward activated, matrix‐remodeling states, and epithelial cells displayed enhanced injury‐repair and EMT‐related features, while TGFB1 was preferentially detected in a GPNMB‐expressing macrophage subset expanded in polyps. Potential epithelial–fibroblast communication was enriched within COLLAGEN and TGF‐β modules, with TGFB1–TGFBR1/TGFBR2 and COL1A1–SDC4/CD44 emerging as candidate ligand–receptor pairs. Human polyps showed collagen deposition, α‐SMA–high fibroblast enrichment, EMT‐like epithelial changes, and nuclear p‐Smad3 accumulation. The mouse model recapitulated this pattern, and dexamethasone partially reversed it at the protein level.

**Discussion:**

Integrating human single‐cell transcriptomics with tissue‐ and protein‐level validation, this study characterizes a coordinated, TGF‐β/Smad3‐associated epithelial–fibroblast remodeling program underlying collagen deposition in CRSwNP. These candidate axes, together with a macrophage source of TGFB1, offer mechanistic clues to epithelial–fibroblast–immune crosstalk; their partial reversibility by dexamethasone supports pathway‐targeted anti‐remodeling strategies, while causal roles await spatial and functional validation.

**Abbreviations**
ANOVA: analysis of variance
BCA: bicinchoninic acid
BSA: bovine serum albumin
CRS: chronic rhinosinusitis
CRSsNP: chronic rhinosinusitis without nasal polyps
CRSwNP: chronic rhinosinusitis with nasal polyps
DAPI: 4′,6‐diamidino‐2‐phenylindole
DEX: dexamethasone
ECM: extracellular matrix
EMT: epithelial–mesenchymal transition
FGSEA: fast gene set enrichment analysis
GEO: Gene Expression Omnibus
GO: Gene Ontology
GSEA: gene set enrichment analysis
HRP: horseradish peroxidase
H&E: hematoxylin and eosin
IL‐1β: interleukin‐1β
KEGG: Kyoto Encyclopedia of Genes and Genomes
MSigDB: Molecular Signatures Database
NES: normalized enrichment score
OVA: ovalbumin
PAS: periodic acid–Schiff
PBS: phosphate‐buffered saline
PCA: principal component analysis
PPI: protein–protein interaction
PVDF: polyvinylidene fluoride
p‐Smad3: phosphorylated Smad3
RIPA: radioimmunoprecipitation assay
scRNA‐seq: single‐cell RNA sequencing
SDS‐PAGE: sodium dodecyl sulfate–polyacrylamide gel electrophoresis
SEB: *Staphylococcus aureus* enterotoxin B
SEM: standard error of the mean
TBST: tris‐buffered saline with Tween‐20
TGF‐β: transforming growth factor beta
TSA: tyramide signal amplification
UMAP: uniform manifold approximation and projection
UMI: unique molecular identifier
α‐SMA: alpha‐smooth muscle actin

## 1 Introduction

Chronic rhinosinusitis with nasal polyps (CRSwNP) is a clinical phenotype of CRS characterized by a high tendency toward recurrence and marked heterogeneity in treatment response; its pathological basis is not limited to persistent inflammatory responses but also includes structural abnormalities such as impaired nasal mucosal barrier function, edema, and polypoid hyperplasia [1, 2, 3, 4]. Long‐term inflammatory stimulation and repeated cycles of injury and repair may drive structural remodeling of the nasal mucosa, allowing these alterations to progressively contribute to the chronic progression, treatment refractoriness, and postoperative recurrence of CRSwNP [3, 5]. Therefore, it is necessary to elucidate the mechanisms underlying CRSwNP progression from the perspective of tissue structural remodeling and its cellular basis.

During nasal mucosal tissue remodeling in CRSwNP, epithelial cells, stromal cells, infiltrating immune cells, and the extracellular matrix (ECM) collectively constitute key components of the dynamically evolving local pathological microenvironment. Epithelial cells maintain mucosal barrier function and may acquire a certain degree of phenotypic plasticity during inflammatory stimulation and injury repair. Stromal cells, especially fibroblasts, are important contributors to extracellular matrix deposition and mucosal structural remodeling. Collagen deposition, as one of the key processes of ECM remodeling, represents an important manifestation of stromal remodeling [6, 7, 8]. In addition to endogenous inflammatory mediators, microbial superantigen‐associated stimulation, such as that related to Staphylococcus aureus enterotoxin B, has also been implicated in CRSwNP‐related mucosal inflammation and polypoid lesion formation [9]. In parallel, infiltrating immune cells have been implicated as important sources of TGF‐β1 in nasal polyps; in particular, macrophages and eosinophils have been reported to be major cellular sources of TGF‐β1 in chronic airway inflammatory diseases [10, 11]. Meanwhile, multiple inflammatory factors and tissue‐remodeling signals may participate in these cellular processes, among which TGF‐β1 and IL‐1β are closely associated with epithelial injury repair and EMT [12, 13]. Our previous study found decreased expression of epithelial barrier–related molecules (especially E‐cadherin, ZO‐1, and Occludin) and increased expression of mesenchymal markers (typified by N‐cadherin, Vimentin, and α‐SMA) in the nasal mucosa, and further indicated that the TGF‐β/Smad and Wnt/β‐catenin pathways are involved in nasal mucosal EMT [14]. *COL1A1* has been reported to regulate nasal epithelial EMT and inflammatory responses through Wnt/β‐catenin signaling, thereby linking *COL1A1*‐mediated matrix remodeling to aberrant epithelial repair and mucosal remodeling in CRSwNP [15]. Although previous studies have revealed the complexity of CRSwNP nasal mucosal remodeling from the perspectives of single‐cell subpopulations and epithelial–stromal interactions [13, 16, 17, 18], the relationships among tissue structural abnormalities, cellular state transitions, and intercellular communication within the overall pathological microenvironment—including the relative contributions of immune and stromal compartments to TGF‐β1 signaling—remain insufficiently investigated.

Against this background, the present study integrated public single‐cell transcriptomic datasets, surgically resected human nasal specimens, and an ovalbumin (OVA)/Staphylococcus aureus enterotoxin B (SEB)‐induced CRSwNP‐like mouse model to investigate epithelial–stromal coordination during nasal mucosal remodeling. This design allowed us to connect cell‐state transitions and inferred intercellular communication with tissue‐level extracellular matrix alterations at both the transcriptomic and protein levels in CRSwNP.

## 2 Methods

### 2.1 Source of Single‐Cell Transcriptomic Data From Intranasal Tissues

We retrieved the human nasal tissue scRNA‐seq datasets GSE235711 and GSE276503 from the Gene Expression Omnibus (GEO). After excluding CRSsNP samples, a total of 41 CRSwNP samples and 7 healthy control samples were included for subsequent analysis [13, 16].

### 2.2 Single‐Cell Transcriptomic Data Processing and Cell Subcluster Classification

Single‐cell data preprocessing, downstream statistical analysis, and visualization were conducted using R software (version 4.5.3). Raw count matrices were converted into Seurat objects with Seurat version 5.4.0, followed by cell‐ and gene‐level quality‐control filtering [19]. Genes expressed in at least 3 cells were retained, and cells were filtered according to detected genes, unique molecular identifier (UMI) counts, mitochondrial gene proportion, and hemoglobin gene proportion using thresholds of 100–6000, 500–30,000, < 30%, and < 1%, respectively. After normalization, selection of 2000 highly variable features, scaling with nFeature_RNA, nCount_RNA, percent. mt, and percent. HB was regressed out, and principal component analysis (PCA) was performed; doublets were removed using DoubletFinder (version 2.0.6), with pN = 0.25 and an expected doublet rate of 4% [20]. The retained singlets were reprocessed and integrated using Harmony [21], followed by clustering based on the first 14 dimensions at a resolution of 0.6. Ambient RNA contamination was assessed using decontX (version 1.8.0), and cells with contamination scores > 0.25 were excluded. The remaining cells were visualized by Uniform Manifold Approximation and Projection (UMAP).

Differentially expressed genes for each cluster were identified by FindAllMarkers and displayed with DotPlot. Epithelial cells and fibroblasts were extracted for secondary clustering. Subclusters were annotated using cluster‐specific differentially expressed genes, canonical lineage markers, and published single‐cell atlases of human nasal mucosa, CRS/CRSwNP, respiratory tissues, and cross‐tissue fibroblasts [13, 16, 17, 22, 23, 24, 25]. Manual annotation integrated marker expression patterns, differential gene profiles, and reference atlas information.

### 2.3 Cell Proportion Statistics and Between‐Group Comparisons

Cell proportions were calculated at the sample level as the percentage of each cell type among total cells. Samples containing fewer than 20 cells were excluded to reduce low‐cell‐number bias. Between‐group differences were assessed using the Wilcoxon rank‐sum test with Benjamini–Hochberg correction and visualized as box plots, bar plots, and stacked proportion plots.

### 2.4 Differential Expression Analysis and Enrichment Analysis

For epithelial, fibroblast, and macrophage subclusters, we performed Control‐to‐CRSwNP comparisons with FindMarkers to screen disease‐associated transcriptional changes. The Wilcoxon rank‐sum method provided the statistical test for each gene. We regarded genes with log_2_FC > 0.322 and adjusted *p* < 0.05 as higher in CRSwNP, and those with log_2_FC < −0.322 and adjusted *p* < 0.05 as lower in CRSwNP. Volcano plots were prepared in ggplot2 (version 4.0.2), with selected labels added by ggrepel (version 0.9.7).

We performed functional enrichment analysis on differentially expressed genes. For Gene Ontology (GO) enrichment analysis, we applied the enrichGO function from the clusterProfiler package (version 4.18.4), specifying org.Hs.eg.db as the background database, and set the ontology parameter to “ALL” to cover biological process, cellular component, and molecular function categories. Kyoto Encyclopedia of Genes and Genomes (KEGG) pathway enrichment analysis was performed with the enrichKEGG function, and the organism was set to human (hsa) [26].

The significance threshold for all enrichment results was set at an adjusted *p* value < 0.05. We performed gene set enrichment analysis (GSEA) with the fgsea package (version 1.36.2) [27, 28]. The gene list was ranked in descending order according to log_2_FC values, which were used as the ranking statistic. Reference gene sets for GSEA were obtained from the MSigDB Human v2026.1.Hs database, comprising Hallmark, C2 curated, and C5 Gene Ontology gene sets [29, 30]. After merging the above GMT files, duplicate entries were removed according to gene set names to construct a custom reference gene set library, which was subsequently used for fgsea analysis. The analysis parameters were set as follows: minimum gene set size, 3; maximum gene set size, 500; and number of permutations, 10,000. Significantly enriched pathways were defined as those with a nominal *p* value < 0.05. For selected key pathways, NES bar plots were generated with ggplot2, and pathway–gene association network construction was performed with igraph (version 2.2.2) and ggraph (version 2.2.2).

### 2.5 Pseudotime Analysis

CytoTRACE2 (version 1.1.0) was applied to evaluate the differentiation potential of cells in each subcluster, and the results were displayed as UMAP distribution plots and violin plots [31]. Pseudotime trajectory analysis was conducted with Monocle3 (version 1.4.26) [32]. The raw RNA count matrix from the Seurat object was used to construct the Monocle3 object, and precomputed low‐dimensional coordinates were imported. Trajectories were visualized in the UMAP space of annotated fibroblasts and epithelial cells. Key genes representing epithelial states and fibroblast differentiation states were selected, and their expression dynamics along the pseudotime axis were plotted with the plot_genes_in_pseudotime function.

### 2.6 Cell–Cell Communication Analysis

Cell–cell communication analysis was performed using the CellChat package (version 1.6.1) [33]. CellChat objects were constructed separately for the Control and CRSwNP groups according to cell‐type annotation results. We used CellChatDB.human as the reference ligand–receptor database. For each group, CellChat first identified expressed signaling genes and overrepresented ligand–receptor pairs. The workflow then incorporated PPI‐based smoothing, estimated intercellular communication probabilities, removed low‐abundance interactions, inferred pathway‐level signaling activity, and summarized the global communication network. Communication probabilities were calculated by the triMean method; PPI network smoothing was performed based on PPI.human, and min.cells was set to 10 to reduce false‐positive interactions caused by low‐abundance cell populations. The two CellChat objects were then merged into a comparison object to compare differences in the number of interactions, interaction strength, and signaling pathway information flow between the Control and CRSwNP groups. Cellular sending and receiving patterns and communication modules were displayed as circle plots, chord plots, bubble plots, and contribution plots.

CellChat analyses focusing on epithelial–macrophage and macrophage–fibroblast interactions were performed using the same workflow and parameters as described above.

### 2.7 Human Nasal Specimens

The clinical study was approved by the Ethics Review Committee of the First Affiliated Hospital of Hunan University of Chinese Medicine (approval No. HN‐LL‐YJSLW‐2026‐25; date of approval: January 1, 2026). As only residual surgical specimens were used and no identifiable personal information was involved, the requirement for written informed consent was waived by the ethics committee. Nasal polyp tissues were obtained from three patients with CRSwNP (one male and two females, aged 18–65 years) undergoing functional endoscopic sinus surgery at the First Affiliated Hospital of Hunan University of Chinese Medicine; CRSwNP was diagnosed according to the European Position Paper on Rhinosinusitis and Nasal Polyps 2020 (EPOS 2020) and the Chinese guideline for the diagnosis and treatment of chronic rhinosinusitis (2018), that is, two or more symptoms (including nasal obstruction and/or nasal discharge) persisting for ≥ 12 weeks, with bilateral polyps in the middle meatus confirmed by nasal endoscopy [2, 34]. Control nasal mucosa (inferior turbinate and/or nasal septal mucosa) was obtained from three patients with nasal septal deviation (two males and one female, aged 18–65 years) without comorbid chronic rhinosinusitis. None of the participants had used any medication within 2 weeks before surgery. Patients who were pregnant or lactating, had severe systemic diseases, had a history of nasal surgical intervention, or had known immunodeficiency or parasitic infection were excluded. All specimens were fixed in 4% paraformaldehyde, embedded in paraffin, and sectioned at 4 μm.

### 2.8 Establishment of the Animal Model

The Experimental Animal Ethics Committee of the First Affiliated Hospital of Hunan University of Chinese Medicine approved the animal protocol on September 1, 2025 (approval No. 202508201). We obtained female BALB/c mice aged 5–6 weeks from Hunan SJA Laboratory Animal Co. Ltd. (Hunan, China). All mice were housed under specific pathogen‐free conditions in an institutionally licensed animal facility [license No. SYXK (Xiang) 2025–0013]. Throughout the experiment, the mice remained in a specific pathogen‐free facility maintained at 24°C, 55%–65% relative humidity, and a 12‐h light/dark cycle. After 1 week of acclimatization, 24 mice were randomly assigned to three groups: the control group (CON), the CRSwNP‐like model group (CRSwNP), and the dexamethasone‐treated group (DEX), with eight mice per group. Within each group, four mice were used for histopathological and immunofluorescence analyses, and the remaining four were used for nasal mucosa collection and Western blot analysis. The study was conducted in accordance with relevant institutional guidelines and regulations and reported in compliance with the ARRIVE guidelines.

The CRSwNP‐like mouse model was established with reference to previously reported OVA/SEB‐induced models of allergic rhinosinusitis with nasal polyp‐like lesions, with appropriate modifications according to the design of the present study [35]. Control mice received PBS using the same route, volume, and schedule as the model group. For CRSwNP‐like modeling, mice received intraperitoneal injections of 200 μL OVA/aluminum hydroxide suspension on Days 0 and 5. Each injection contained 25 μg ovalbumin (OVA; Shanghai Yuanye Bio‐Technology Co. Ltd., Shanghai, China; Cat. No. S12015; CAS No. 9006‐59‐1; ≥ 99%) and 2 mg aluminum hydroxide. From Days 12 to 18, the model mice were challenged intranasally with 3% OVA once daily at 40 μL per mouse. The same dose was then given three times per week from Days 19 to 103. From Days 47 to 103, Staphylococcal enterotoxin B (SEB; Toxin Technology, Cat. No. BT202, Lot No. 92815B) was administered intranasally once weekly at 10 ng per mouse in 20 μL PBS, in addition to OVA stimulation. From Day 104 to Day 131, mice in the DEX group received intraperitoneal injections of dexamethasone sodium phosphate (1 mg/kg, once daily for 4 consecutive weeks; Sinopharm Rongsheng Pharmaceutical Co. Ltd., China), while mice in the CON and CRSwNP groups received an equal volume of pure water via the same route.

### 2.9 Histopathological Analysis and Immunofluorescence Staining

After isoflurane anesthesia, mice received transcardial perfusion, and nasal tissues were collected for histological analysis. The samples were fixed, dehydrated, embedded in paraffin, and cut into 4‐μm serial sections. We used H&E staining to examine nasal mucosal architecture, inflammatory infiltration, and polypoid changes. Sirius Red staining was applied to evaluate collagen deposition. Briefly, deparaffinized and rehydrated sections were stained with Sirius Red solution for 8 min, rapidly dehydrated in absolute ethanol, cleared in xylene, and mounted with neutral balsam. Collagen fibers appeared red under bright‐field microscopy. Their distribution was further examined under polarized light using an upright microscope.

We used a tyramide signal amplification (TSA)‐based immunofluorescence method to detect target proteins in paraffin‐embedded nasal tissue sections. After dewaxing, graded rehydration, heat‐induced antigen retrieval, endogenous peroxidase blocking, and serum blocking, sections were incubated with primary antibodies and HRP‐conjugated secondary antibodies. TYR‐488 and TYR‐555 were used for two‐color signal development, with an additional antigen retrieval step between the two staining cycles. In mouse nasal tissues, double immunofluorescence staining was performed for E‐cadherin/phosphorylated Smad3 and COL1A1/α‐SMA, and single immunofluorescence staining was performed for N‐cadherin and POSTN. The same protocol was applied to human nasal tissue sections with the following combinations: E‐cadherin/phosphorylated Smad3, COL1A1/α‐SMA, and N‐cadherin/POSTN. The primary antibodies included anti‐E‐cadherin (Proteintech, 20874‐1‐AP, 1:500), anti‐phosphorylated Smad3 (Abways, CY5140, 1:500), anti‐COL1A1 (Selleck, F1228, 1:500), anti‐α‐SMA (Servicebio, GB111364, 1:800), anti‐N‐cadherin (Servicebio, GB11135, 1:500), and anti‐periostin (Proteintech, 66491‐1‐Ig, clone 1A11A3, 1:4000). Primary antibody dilutions were optimized for the TSA‐based signal amplification system. After DAPI counterstaining and anti‐fade mounting, sections were scanned using a digital slide scanner (LG‐FS80, Pinuofei Biotechnology, China). Images acquired under identical settings were used for histological assessment and immunofluorescence quantification.

### 2.10 Masson's Trichrome and PAS Staining of Human Specimens

Paraffin sections of human nasal tissues were stained with Masson's trichrome to evaluate subepithelial collagen deposition and with periodic acid–Schiff (PAS) to assess goblet cell hyperplasia and mucus production, following standard protocols. Stained sections were scanned with a digital slide scanner for qualitative histological assessment.

### 2.11 Western Blot Analysis

Total protein was extracted from mouse nasal mucosa using Radioimmunoprecipitation assay (RIPA) lysis buffer supplemented with protease and phosphatase inhibitors, and protein concentrations were determined with a Bicinchoninic acid (BCA) assay. Equal amounts of protein were separated by 8%–10% Sodium dodecyl sulfate–polyacrylamide gel electrophoresis (SDS‐PAGE) and transferred onto Polyvinylidene fluoride (PVDF) membranes (Millipore). After blocking with Bovine serum albumin (BSA) in Tris‐buffered saline with Tween‐20 (TBST), membranes were incubated overnight at 4°C with primary antibodies against COL1A1 (Affinity, AF7001, 1:500), POSTN (Affinity, DF6746, 1:500), N‐cadherin (Affinity, AF5239, 1:500), α‐SMA (Proteintech, 67735‐1‐Ig, 1:20000), E‐cadherin (Affinity, BF3043, 1:2000), phosphorylated Smad3 (Affinity, BF3080, 1:1000), Smad3 (Affinity, BF3079, 1:1000), and GAPDH (Proteintech, 60004‐1‐Ig, 1:10000), followed by incubation with HRP‐conjugated secondary antibodies (1:5000) at room temperature. Signals were developed with enhanced chemiluminescence reagent and captured using a ChemiScope 6100 imaging system (Clinx, Shanghai, China). Band intensities were quantified by densitometry and normalized to GAPDH; p‐Smad3/Smad3 ratios were additionally calculated.

### 2.12 Statistical Analysis

Single‐cell RNA‐seq data were analyzed using Seurat, CellChat, Monocle3, CytoTRACE2, clusterProfiler, and fgsea. Default parameters were used unless otherwise specified. Differentially expressed genes were filtered according to the indicated adjusted *p* value and log_2_ fold‐change thresholds. Sample‐level cell‐type proportions were compared using the Wilcoxon rank‐sum test, with Benjamini–Hochberg correction applied where applicable.

For the animal experiments, the individual mouse was considered the experimental unit and an independent biological replicate. Within each group, four mice were used for histopathological and immunofluorescence analyses and four for Western blot analysis (*n* = 4 biological replicates per assay per group). For histopathological and immunofluorescence measurements, three to four microscopic fields were obtained from each mouse and averaged to generate a single value for that animal before statistical analysis. No animals or data points were excluded from the final analyses. The exact sample size for each analysis is reported in the corresponding figure legends. Animal data are presented as the mean ± SEM. Epithelial thickness, Sirius Red–positive area, and immunofluorescence measurements were compared between the CON and CRSwNP groups using two‐tailed unpaired *t* tests with Welch's correction; polypoid lesion counts and epithelial pathology scores were analyzed using two‐tailed Mann–Whitney *U* tests. For Western blot, band intensities were normalized to GAPDH, with p‐Smad3 additionally normalized to total Smad3, and compared among the CON, CRSwNP, and DEX groups using ordinary one‐way analysis of variance (ANOVA) followed by Tukey's multiple comparisons test.

For human specimens, three microscopic fields were randomly selected from each sample, and the measurements were averaged and normalized to the mean of the control group. Between‐group comparisons (CRSwNP vs. control) were performed using two‐tailed unpaired *t* tests with Welch's correction (*n* = 3 per group), and exact *p* values are reported. Masson's trichrome and PAS staining were assessed qualitatively.

Statistical analyses of experimental data were performed using GraphPad Prism (version 9.0.0, GraphPad Software, San Diego, CA, USA). *p* < 0.05 was considered statistically significant.

## 3 Results

### 3.1 Single‐Cell Transcriptomics Reveals the Major Cellular Composition of Intranasal Tissues

We integrated published single‐cell RNA sequencing (scRNA‐seq) data from intranasal tissue samples, including ethmoid sinus mucosa, nasal polyps, middle turbinate, and inferior turbinate tissues [13, 16]. The seven control samples were mainly derived from inferior turbinate and ethmoid sinus mucosa, while the 41 CRSwNP samples were derived from inferior turbinate, middle turbinate, ethmoid sinus mucosa, and nasal polyps. Given the potential intrinsic differences among tissues from distinct anatomical sites, tissue‐source heterogeneity was taken into consideration in the analysis, and the cellular composition of CRSwNP was mainly analyzed at the level of the overall intranasal tissue microenvironment. Following quality control and preprocessing (Supporting Information S1: Figures S1–S4), unsupervised clustering and manual annotation identified 13 major cell populations (Figure 1a,b): T cells, fibroblasts, endothelial cells, epithelial cells, pericytes, myeloid cells, B cells, mast cells, plasma cells, keratinocytes, plasmacytoid dendritic cells, and Type 1 and Type 2 conventional dendritic cells (cDC1 and cDC2).

**Figure 1:**
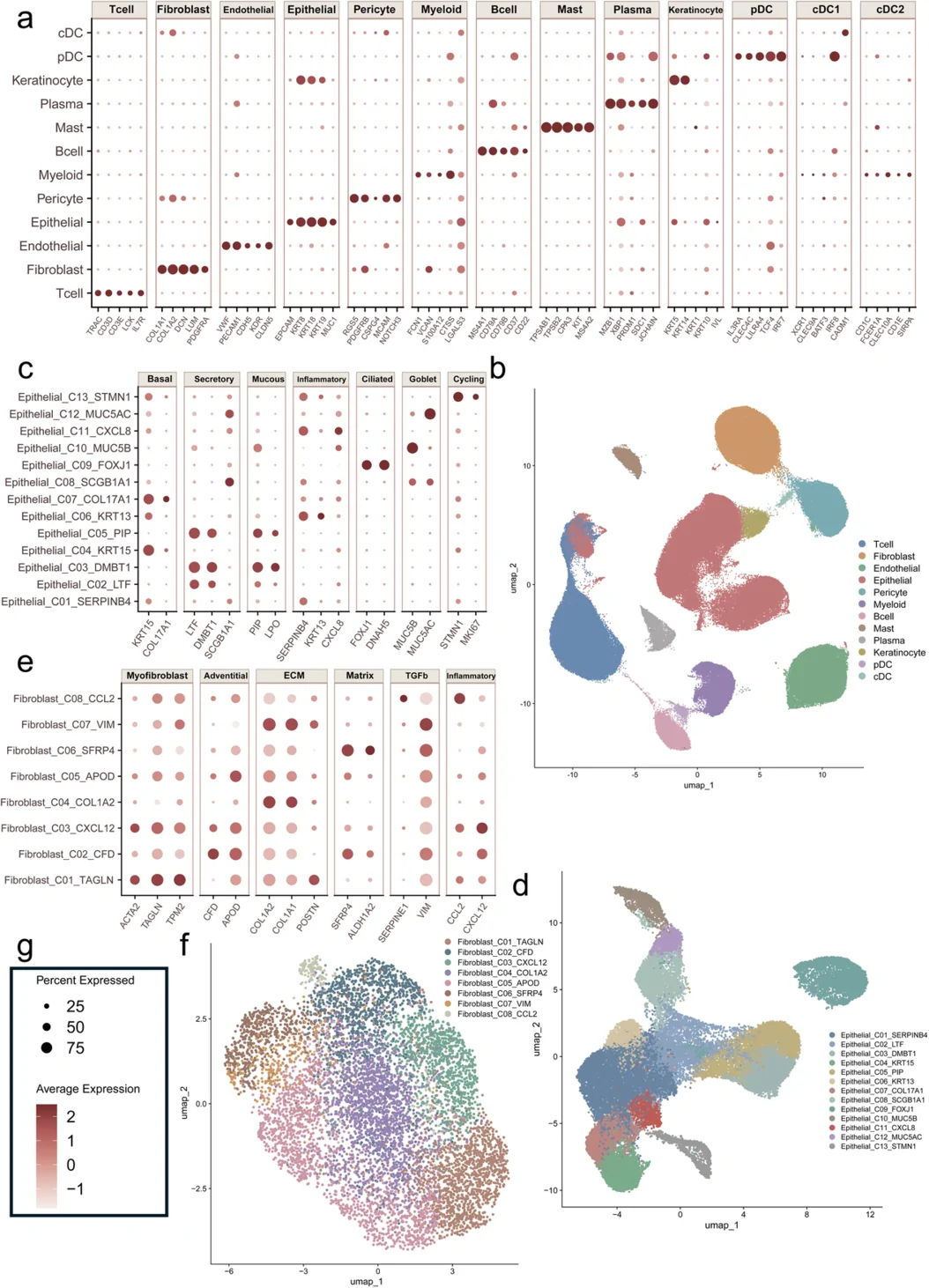
Single‐cell transcriptomic atlas of human nasal tissues with subcluster annotation of epithelial cells and fibroblasts. (a) Dot plot of canonical marker genes across the 13 major cell populations; dot size indicates the percentage of cells expressing the gene, and color indicates scaled average expression. (b) UMAP visualization of the 13 annotated major cell populations. (c, d) Marker‐gene dot plot (c) and UMAP visualization (d) of the 13 epithelial subpopulations, grouped by functional state (basal, secretory, mucous, inflammatory, ciliated, goblet, cycling). (e, f) Marker‐gene dot plot (e) and UMAP visualization (f) of the eight fibroblast subpopulations, grouped by functional state (myofibroblast, adventitial, ECM‐remodeling, matrix‐producing, TGF‐β‐responsive, inflammatory). (g) Shared legend for dot size and color scale in (a, c, e). Canonical marker genes used for annotation are provided in Supporting Information S1: Tables S1–S3.

### 3.2 Heterogeneity of Epithelial and Fibroblast Subpopulations in CRSwNP

After reclustering, epithelial cells were resolved into 13 subpopulations and fibroblasts into 8 subpopulations. Marker‐based annotation identified epithelial states related to basal progenitor, secretory/glandular, inflammatory, ciliated, goblet/mucous, and proliferative phenotypes, as well as fibroblast states related to quiescent adventitial‐like, ECM‐remodeling/matrix‐producing, TGF‐β‐responsive, myofibroblast‐like, and inflammatory phenotypes (Figure 1c–f) [18, 25].

At the sample level, both compartments showed marked compositional heterogeneity in CRSwNP (Figure 2). Shifts within fibroblasts were relatively modest (Figure 2a): inflammatory *CCL2* ^+^ fibroblasts were increased in CRSwNP (*p* = 0.015) and *APOD* ^+^ fibroblasts were reduced (*p* = 0.013), whereas the myofibroblast‐like *TAGLN* ^+^ and matrix‐producing *COL1A2* ^+^ subsets remained comparable between groups (*p* = 0.16 and *p* = 1, respectively). The epithelial compartment showed more pronounced changes, with 6 of the 13 subpopulations differing significantly between groups (Figure 2b): the inflammatory *CXCL8* ^+^ subset was expanded (*p* = 0.023), together with *MUC5AC* ^+^ goblet cells (*p* = 0.0038), whereas homeostatic *FOXJ1* ^+^ ciliated cells were reduced (*p* = 0.0013); the *LTF* ^+^, *KRT15* ^+^, and *COL17A1* ^+^ subsets also differed significantly (*p* = 0.014, *p* = 0.015, and *p* < 0.001, respectively). Thus, while fibroblast composition shifted only modestly, epithelial subpopulations tilted toward inflammatory and secretory states at the expense of homeostatic ciliated cells.

**Figure 2:**
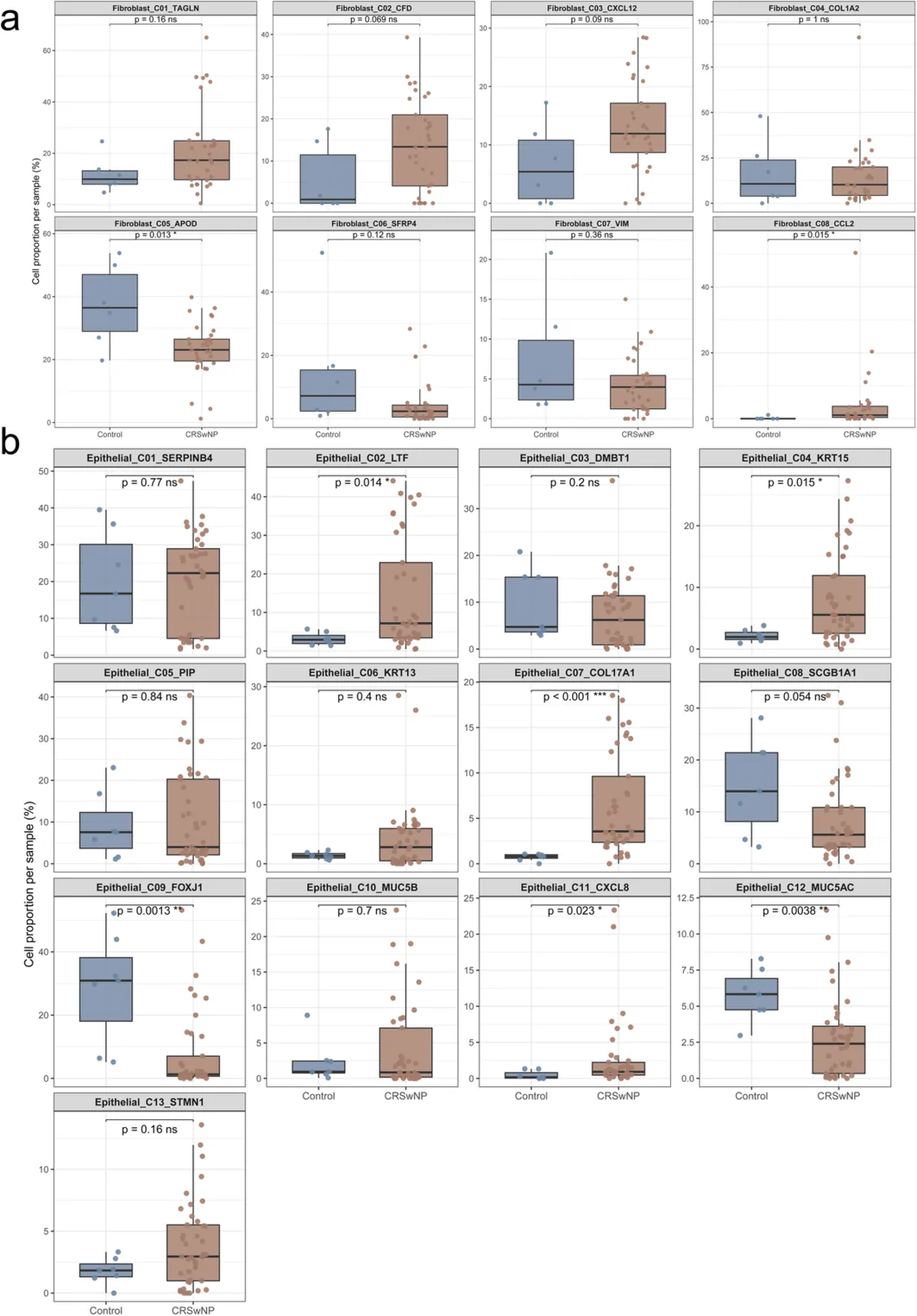
Sample‐level compositional shifts of epithelial and fibroblast subpopulations in CRSwNP. (a) Box plots comparing the proportions of the eight fibroblast subpopulations between the Control and CRSwNP groups. (b) Box plots comparing the proportions of the 13 epithelial subpopulations between groups. Proportions were calculated per sample, and each dot represents one sample. Exact *p* values are shown above each comparison (two‐sided Wilcoxon rank‐sum test with Benjamini–Hochberg correction). ns, not significant; *p < 0.05, **p < 0.01, ***p < 0.001.

Reclustering of the myeloid compartment resolved five macrophage subpopulations (Supporting Information S1: Figure S10a,b), spanning *C1QA* ^+^ resident, *IL1B* ^+^/*NFKBIA* ^+^/*CXCL8* ^+^ inflammatory and monocyte‐like, and *GPNMB* ^+^ remodeling states (the latter co‐expressing SPP1, TREM2 and MMP9). Notably, TGFB1 was preferentially detected in the *GPNMB* ^+^ subset. Only this subset differed between groups, being more abundant in CRSwNP (*p *= 0.025), whereas the remaining subsets were comparable (all *p* > 0.05; Supporting Information S1: Figure S10c).

### 3.3 Epithelial Cells and Fibroblasts in CRSwNP Exhibit Features of Inflammatory Activation and Matrix Remodeling

To investigate the transcriptional changes of epithelial cells and fibroblasts in the CRSwNP microenvironment, we performed differential expression analysis of epithelial cells and fibroblasts between the CRSwNP and Control groups, followed by pre‐ranked GSEA to evaluate changes in related functional pathways.

In epithelial cells, the volcano plot revealed extensive differential gene expression in the CRSwNP group compared with the Control group (Figure 3a). Among them, genes associated with epithelial injury responses, basal‐like cell states, epithelial repair, and tissue remodeling were upregulated, with increased expression of *SERPINB4, KRT13, KRT15, COL17A1, MMP7*, and *VIM*. In contrast, genes involved in maintaining secretory cell function, ciliary differentiation, and mucosal barrier homeostasis, such as *SCGB1A1, FOXJ1, BPIFA1*, and *MUC5B*, were downregulated.

**Figure 3:**
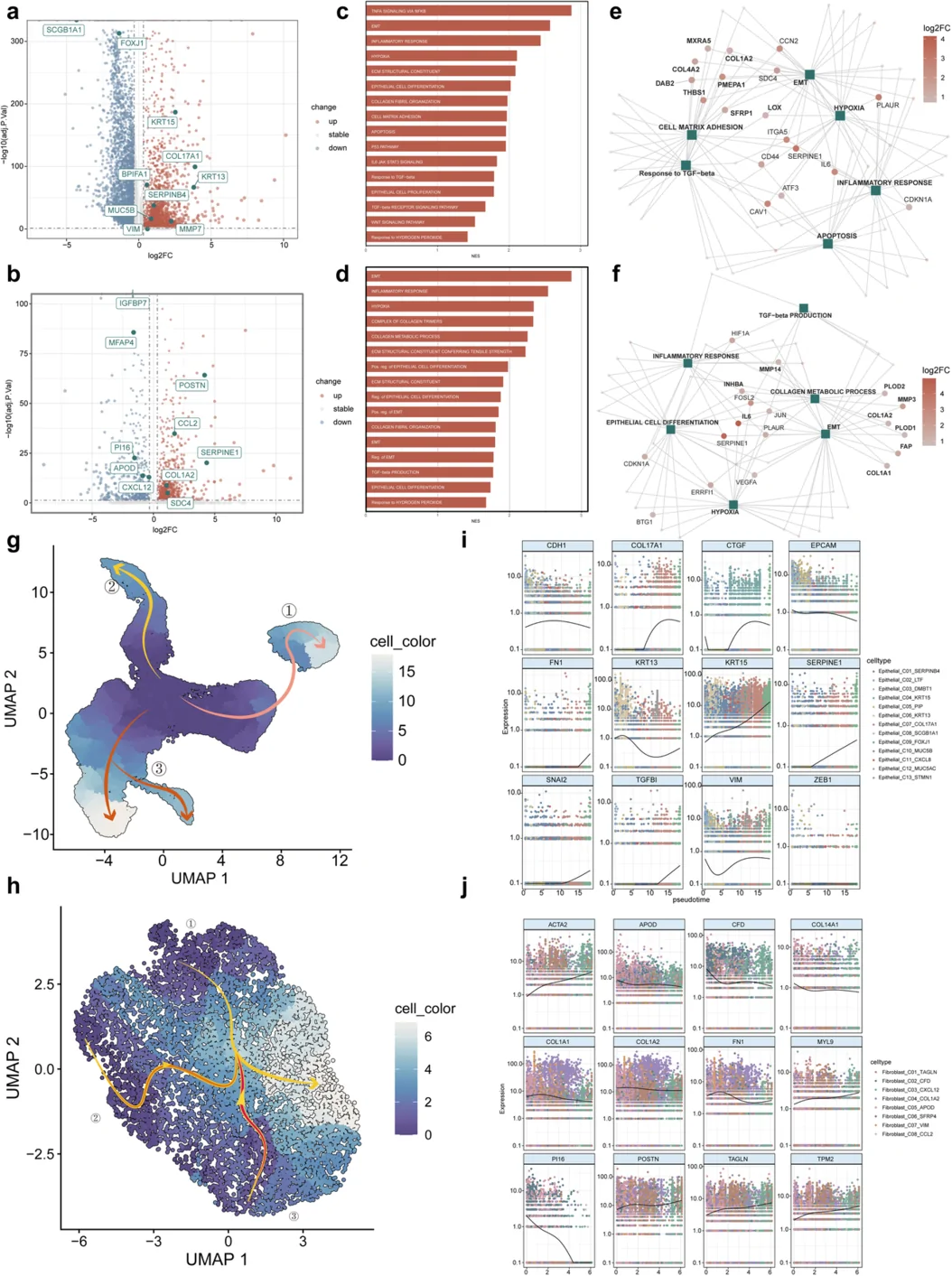
Transcriptional reprogramming and pseudotime dynamics of epithelial cells and fibroblasts in CRSwNP. (a, b) Volcano plots of differentially expressed genes in epithelial cells (a) and fibroblasts (b) (CRSwNP vs. Control; two‐sided Wilcoxon rank‐sum test; thresholds |log_2_FC | > 0.322 and adjusted *p* < 0.05). (c, d) GSEA of epithelial cells (c) and fibroblasts (d); bars show representative enriched pathways, with the *x*‐axis indicating the normalized enrichment score (NES). (e, f) Pathway–gene networks of representative enriched pathways in epithelial cells (e) and fibroblasts (f); square nodes represent pathways, circular nodes represent genes (color, log_2_FC), and edges indicate pathway–gene membership. (g, h) Monocle3 pseudotime trajectories of epithelial cells (g) and fibroblasts (h) in UMAP space. Numbered arrows (①–③) indicate the inferred directions of cell‐state progression; trajectory roots were assigned according to CytoTRACE2‐inferred differentiation potential and subcluster marker profiles (Supporting Information S1: Figures S5–S8). (i, j) Expression dynamics of representative state markers along pseudotime in epithelial cells (i) and fibroblasts (j); dots represent single‐cell expression levels, and curves represent fitted trends. Complete differential‐expression lists and FGSEA results are provided in Supporting Information S1: Tables S4–S7.

Similarly, differential expression analysis of fibroblasts revealed disease‐associated gene expression features (Figure 3b). In fibroblasts from the CRSwNP group, *POSTN, CCL2, SERPINE1, COL1A2*, and *SDC4* were upregulated, suggestive of biological processes related to matrix deposition, inflammatory chemotaxis, TGF‐β responses, collagen formation, and cell–matrix interactions. Meanwhile, *PI16, APOD, CXCL12, MFAP4*, and *IGFBP7*, which are associated with homeostatic regulation, matrix structural stability, and regulation of the tissue microenvironment, were downregulated [7]. Overall, the transcriptional alterations in fibroblasts in CRSwNP support their potential roles in inflammatory amplification, collagen deposition, and stromal remodeling.

To clarify the functional pathways associated with these differentially expressed genes, we performed GSEA enrichment analysis of epithelial cells and fibroblasts (Figure 3c,d). In epithelial cells, pathways related to inflammatory response, EMT, hypoxia, epithelial cell differentiation, extracellular matrix organization, TGF‐β/WNT signaling, and oxidative stress were markedly enriched, consistent with inflammatory activation, altered differentiation, and enhanced remodeling signals in CRSwNP epithelial cells. In fibroblasts, the enriched pathways told a similar story: EMT, inflammatory response, hypoxia, collagen metabolism, and TGF‐β production all featured prominently. Such a profile fits a stromal compartment primed for matrix deposition in an inflamed setting.

After integrating GO, KEGG, and GSEA enrichment results to construct pathway–gene networks, multiple enriched pathways shared recurrent genes (Figure 3e,f), with IL6 and SERPINE1 common to both cell types, CCN2 and SDC4 recurrent in the epithelial network, and INHBA and COL1A1 recurrent in the fibroblast network. These genes are involved in pro‐inflammatory factor release, TGF‐β signaling, fibrotic responses, extracellular matrix deposition, and cell–matrix adhesion, suggesting that epithelial cells and fibroblasts in CRSwNP may jointly shape an abnormal local microenvironment through inflammatory signaling, TGF‐β responses, and ECM remodeling programs [24, 36, 37, 38].

### 3.4 Epithelial EMT and Fibroblast Activation in the CRSwNP Tissue Microenvironment

We next applied Monocle3 to reconstruct pseudotime trajectories of epithelial and fibroblast subpopulations. CytoTRACE2 analysis was used to infer the relative differentiation potential of these cells, with higher CytoTRACE2 scores supporting less‐differentiated or progenitor‐like states (Supporting Information S1: Figures S5–S8). Based on subcluster‐specific marker profiles and CytoTRACE2‐inferred differentiation potential, *LTF/DMBT1/SERPINB4*‐high epithelial cells and *CFD/APOD* fibroblasts were assigned as the respective trajectory roots. The epithelial trajectory diverged from the starting region toward three major directions: *FOXJ1^+^* ciliated epithelial cells, *SCGB1A1^+^/MUC5B^+^/MUC5AC^+^* secretory‐goblet/mucous states, and *KRT15^+^/COL17A1^+^/KRT13^+^/CXCL8^+^/STMN1^+^* injury‐repair and remodeling‐associated states. The fibroblast trajectory started from relatively quiescent *CFD/APOD* fibroblasts and extended toward *CXCL12^+^* matrix‐regulatory, *COL1A2^+^* ECM‐remodeling, and *TAGLN^+^/VIM^+^/CCL2^+^* activated fibroblast states, suggesting progressive stromal activation and matrix remodeling(Figure 3g,h).

To further characterize the expression dynamics of differentiation‐associated marker genes across cell subpopulations in the pseudotime context, dynamic gene expression analysis was performed (Figure 3i,j). In epithelial subpopulations, epithelial homeostasis‐related genes, such as *EPCAM* and *CDH1*, were generally downregulated along pseudotime; in contrast, *KRT15* and *COL17A1* increased continuously along pseudotime. Meanwhile, *SERPINE1, CTGF*, and *TGFBI* were enriched at the late stage of the trajectory, and *SNAI2* increased at later pseudotime points, suggesting a tendency toward matrix‐remodeling‐associated epithelial changes. The dynamic patterns of *VIM* and *KRT13* further suggested that epithelial cells did not follow a single linear differentiation route. In fibroblast subpopulations, relatively homeostatic markers, including *APOD, CFD*, and *PI16*, gradually decreased along pseudotime. Conversely, *POSTN, TAGLN, TPM2*, and *MYL9* increased continuously, indicating progressive acquisition of activated and contractile phenotypes. In parallel, *COL1A1, COL1A2*, and *FN1* remained highly expressed or further increased during the middle‐to‐late stages of the trajectory, suggesting a transition toward more active ECM‐remodeling functions. Together, these findings suggest loss of homeostatic features in both epithelial cells and fibroblasts, accompanied by increased matrix‐remodeling programs, fibroblast activation, and EMT‐like epithelial changes along pseudotime [7, 39].

### 3.5 Predicted COLLAGEN and TGF‐β Ligand–Receptor Signaling Among Epithelial, Fibroblast, and Macrophage Subpopulations in CRSwNP

CellChat was used to compare ligand–receptor‐level communication between epithelial cells and fibroblasts in the Control and CRSwNP groups. Compared with the Control group, the CRSwNP group was characterized by a greater number of inferred intercellular interactions, higher overall interaction strength, and increased information flow activity of remodeling‐associated pathways, particularly COLLAGEN and TGF‐β, together with LAMININ, FN1, and PERIOSTIN (Figure 4a,g,i). Consistently, heatmap analysis of incoming and outgoing signaling patterns showed altered communication programs across epithelial and fibroblast subpopulations in CRSwNP, with prominent involvement of ECM‐remodeling‐related pathways (Supporting Information S1: Figure S9). Representative subnetworks further highlighted *TAGLN^+^, COL1A2^+^*, and *VIM^+^* fibroblasts as prominent contributors to the enhanced communication network in CRSwNP (Figure 4b).

**Figure 4:**
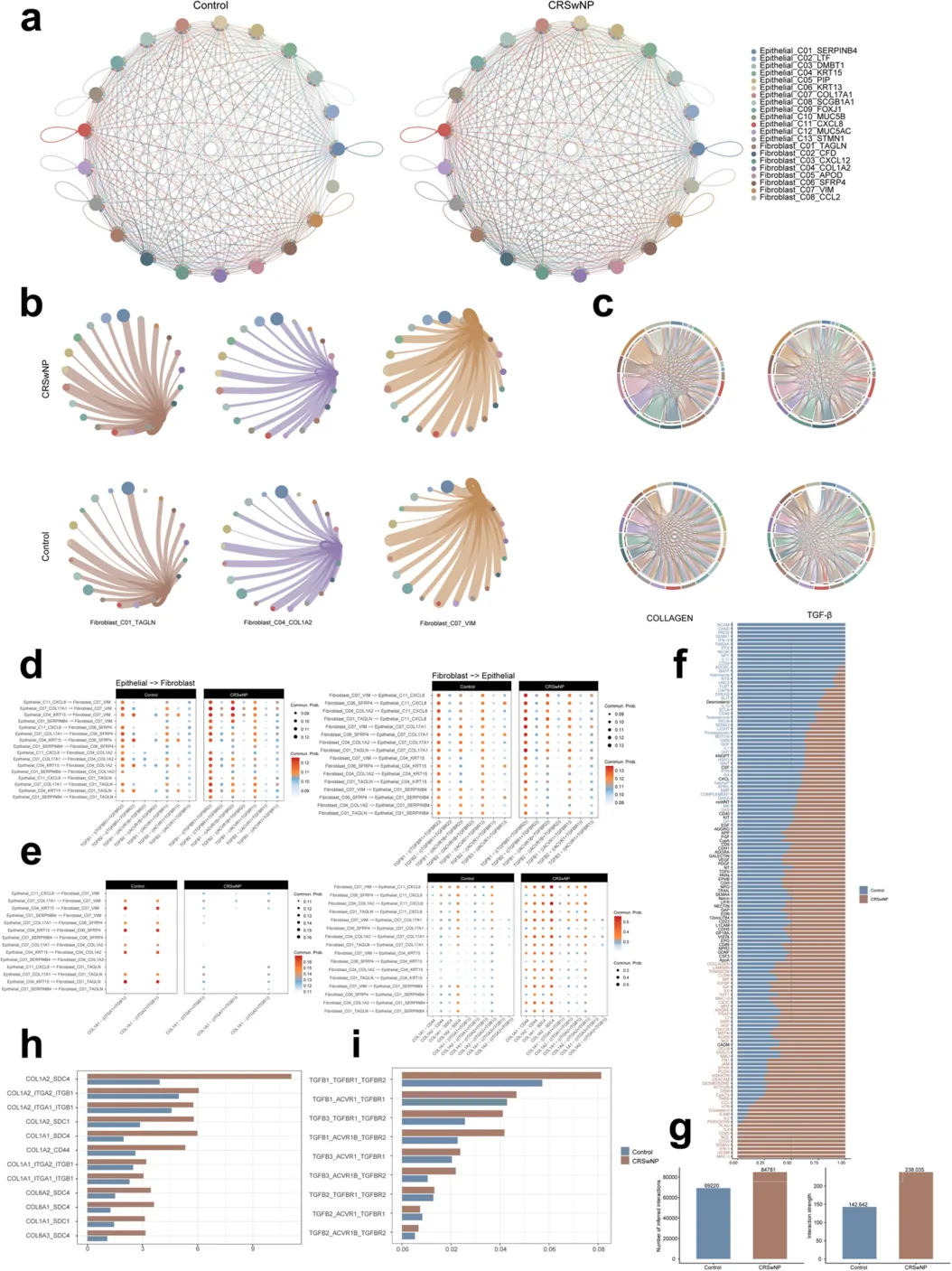
Inferred epithelial–fibroblast communication in CRSwNP. (a) Circle plots of the overall communication networks among epithelial and fibroblast subpopulations in the Control and CRSwNP groups; edge width represents inferred communication probability. (b) Communication networks of representative fibroblast subpopulations (TAGLN^+^, COL1A2^+^, and VIM^+^ fibroblasts), selected on the basis of their sender contributions to the COLLAGEN and TGF‐β pathways. (c) Chord diagrams of the COLLAGEN and TGF‐β networks in the two groups. (d, e) Bubble plots of ligand–receptor interactions in the TGF‐β (d) and COLLAGEN (e) modules, shown in both epithelial‐to‐fibroblast and fibroblast‐to‐epithelial directions; dot color indicates communication probability and dot size indicates statistical significance. (f) Relative information flow of signaling pathways between the two groups. (g) Total number and overall strength of inferred interactions in the two groups. (h, i) Contribution of major ligand–receptor pairs to the COLLAGEN (h) and TGF‐β (i) networks; the *x*‐axis represents summed communication probability. Complete CellChat results are provided in Supporting Information S1: Tables S8 and S9; incoming and outgoing signaling patterns are shown in Supporting Information S1: Figure S9.

Together, the differential expression, functional enrichment, and CellChat information flow analyses pointed to enhanced COLLAGEN and TGF‐β communication activity in the CRSwNP group. Based on previous studies of TGF‐β/Smad signaling, ECM remodeling, and EMT in CRSwNP, the present study focused on the collagen (COLLAGEN) and TGF‐β (TGFb) communication modules (Figure 4c–f,h) [38, 39]. The collagen communication network was denser in the CRSwNP group (Figure 4c), and signals from several activated fibroblast subpopulations, characterized by *TAGLN^+^, COL1A2^+^, SFRP4^+^*, and *VIM^+^* fibroblasts, to specific epithelial subpopulations, marked by *SERPINB4^+^, KRT15^+^, COL17A1^+^*, and *CXCL8^+^* epithelial cells, were markedly enhanced, and the network was attributable to collagen‐related ligand–receptor pairs, especially *COL1A1/COL1A2–CD44, COL1A1/COL1A2–SDC4, COL1A1/COL1A2–ITGA1/ITGB1*, and *COL1A1/COL1A2–ITGA2/ITGB1* (Figure 4e,h). In the TGF‐β cell–cell communication network, multiple fibroblast and epithelial subpopulations appeared to signal reciprocally, mainly involving the canonical ligand–receptor pairs *TGFB1–TGFBR1/TGFBR2, TGFB1–ACVR1/TGFBR1*, and *TGFB3–TGFBR1/TGFBR2* (Figure 4d,f). These results suggested that the collagen communication module mainly originates from activated fibroblasts and acts directionally on specific epithelial subpopulations, with cell–matrix interactions as its major manifestation. In contrast, the TGF‐β network appeared more distributed, with bidirectional signaling inferred between the two compartments. The differences in signaling directionality between these two modules are compatible with distinct but potentially complementary roles in CRSwNP tissue remodeling.

*TGFB1* was preferentially detected in the *GPNMB* ^+^ macrophage subset (Supporting Information S1: Figure S10a); we therefore extended the ligand–receptor analysis to myeloid subpopulations (Supporting Information S1: Figure S10d–h). Macrophage subpopulations—most notably *GPNMB* ^+^ remodeling macrophages—emerged as additional *TGFB1* senders toward fibroblast and epithelial subpopulations, mainly via *TGFB1*–*TGFBR1*/*TGFBR2* and *TGFB1*–*ACVR1*/*TGFBR1*, with reciprocal epithelial–macrophage and fibroblast–macrophage signaling also inferred. These observations nominate remodeling‐associated macrophages as a candidate immune‐cell source of TGF‐β1 in the polyp microenvironment.

Overall, the COLLAGEN and TGF‐β signaling modules may constitute important regulatory axes linking activated fibroblasts with abnormal epithelial states. Based on these findings, subsequent animal experiments were focused on validating the predicted ligand–receptor axes and their corresponding pathological phenotypes.

### 3.6 Protein‐Level Evidence of TGF‐β/Smad‐Associated Epithelial–Fibroblast Remodeling in Human Nasal Polyps

To test whether the computationally inferred remodeling program is reflected at the protein level, we next examined nasal polyp tissues from CRSwNP patients (*n* = 3) and control nasal mucosa (*n* = 3). Masson's trichrome staining showed denser subepithelial collagen deposition in polyps than in control mucosa, and PAS staining revealed more prominent epithelial goblet cell hyperplasia (Figure 5g,h; qualitative assessment). Double immunofluorescence staining lent further support. In the polyp lamina propria, COL1A1 and α‐SMA signals were both markedly stronger than in controls (Figure 5a,b; COL1A1, *p* = 0.0039; α‐SMA, *p* = 0.0438), pointing to fibroblast activation with concurrent collagen accumulation. The epithelium, for its part, showed signs of TGF‐β/Smad3 engagement together with an EMT‐like shift: nuclear p‐Smad3 accumulated in polyp epithelial cells, E‐cadherin was attenuated, and N‐cadherin—weak in control epithelium—became more prominent (Figure 5c–f; p‐Smad3, *p* = 0.0014; E‐cadherin, *p* = 0.0378; N‐cadherin, *p* = 0.0330). POSTN staining was likewise intensified along the epithelial–stromal interface (Figure 5e,f; *p* = 0.0126). As consecutive sections were stained, signals could be compared across markers within corresponding tissue regions: areas of epithelium rich in nuclear p‐Smad3 neighbored stromal regions enriched for COL1A1, α‐SMA and POSTN on adjacent sections, a spatial juxtaposition compatible with the predicted epithelial–fibroblast communication. The distribution of the two matrix proteins was equally informative: COL1A1 and POSTN formed a compact, continuous band beneath the epithelium in control mucosa, but in polyps this band appeared loose and discontinuous, with both signals extending diffusely into the lamina propria—suggesting that matrix remodeling in CRSwNP involves redistribution and loss of organized subepithelial architecture, not merely increased production. Together, these protein‐level observations in human specimens mirror the epithelial injury–repair shift and stromal matrix deposition inferred from the single‐cell analyses (Figure 5b,d,f).

**Figure 5:**
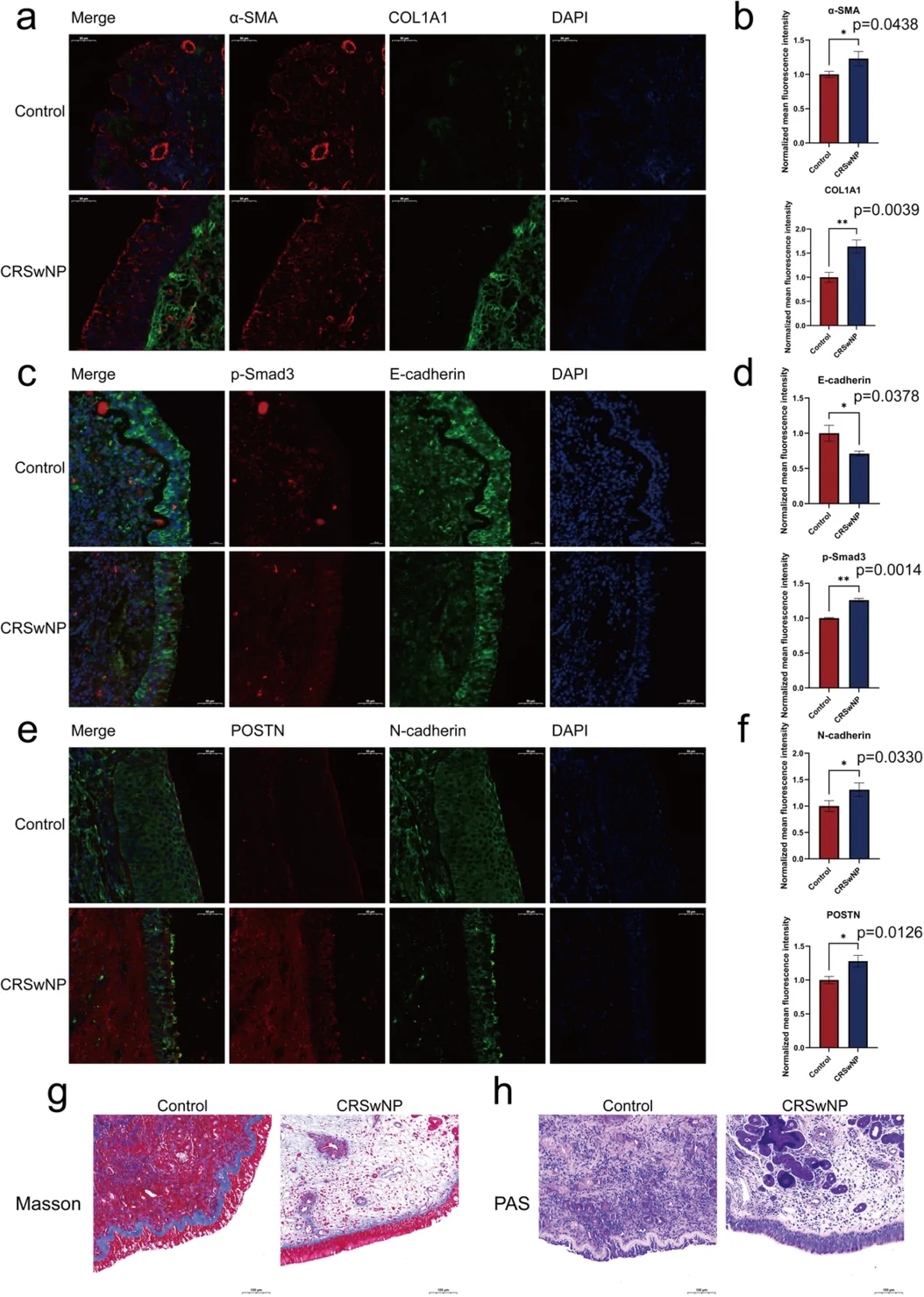
Protein‐level validation of epithelial–fibroblast remodeling in human nasal polyps. (a) Representative TSA‐based double immunofluorescence staining of α‐SMA (red) and COL1A1 (green) in control nasal mucosa and nasal polyps; nuclei were counterstained with DAPI (blue). (b) Quantification of normalized mean fluorescence intensity of α‐SMA (*p *= 0.0438) and COL1A1 (*p *= 0.0039) in the lamina propria. (c) Double immunofluorescence of E‐cadherin (green) and p‐Smad3 (red). (d) Quantification of epithelial E‐cadherin (*p *= 0.0378) and nuclear p‐Smad3 (*p *= 0.0014). (e) Double immunofluorescence of POSTN (red) and N‐cadherin (green). (f) Quantification of N‐cadherin (*p *= 0.0330) and POSTN (*p *= 0.0126). (g) Masson's trichrome staining showing subepithelial collagen deposition (blue). (h) Periodic acid–Schiff (PAS) staining showing goblet‐cell hyperplasia and mucus production. For each sample, three microscopic fields were averaged and normalized to the control‐group mean; *n* = 3 per group; two‐tailed unpaired *t* test with Welch's correction; data are presented as mean ± SEM. *p < 0.05, **p < 0.01. Scale bars = 50 μm. Source data are provided in Supporting Information S1: Table S16.

### 3.7 OVA/SEB Induces CRSwNP‐Like Nasal Mucosal Lesions and Tissue Remodeling in Mice

In this study, an OVA/SEB‐induced CRSwNP‐like mouse model was established [35, 40]. Pathological observations focused mainly on changes in the epithelial layer and the underlying lamina propria within the respiratory mucosal region of the nasal cavity. H&E staining showed marked disruption of nasal mucosal architecture in CRSwNP mice compared with controls, with thickening of the respiratory epithelium (*p *< 0.0001), impaired epithelial continuity, and polypoid protrusions accompanied by inflammatory cell infiltration (Figure 6a). The increased number of polypoid lesions (*p *= 0.0286) and elevated epithelial pathology scores (*p *= 0.0286) indicated successful induction of CRSwNP‐like mucosal hyperplasia and remodeling (Figure 6b–d).

**Figure 6:**
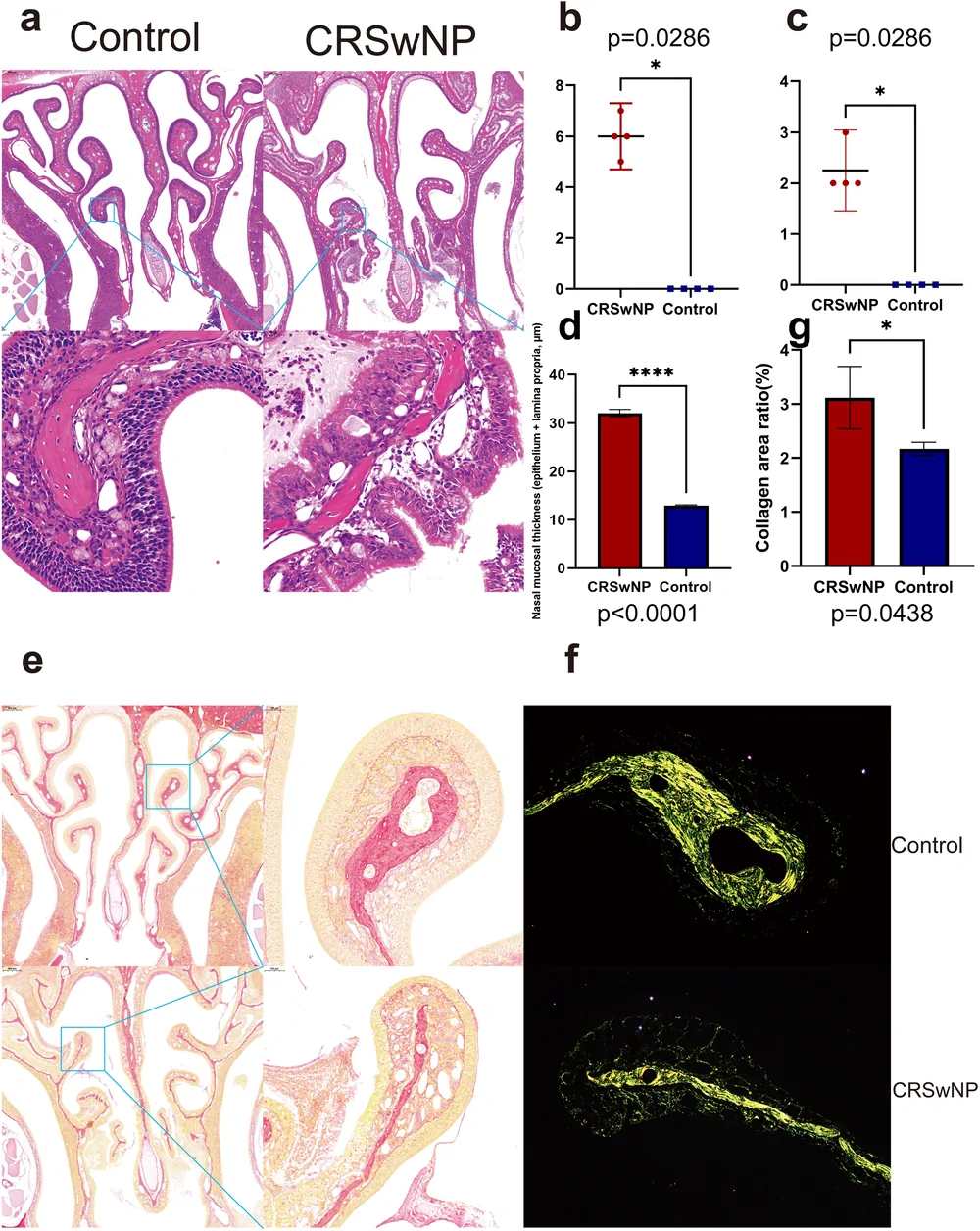
OVA/SEB‐induced CRSwNP‐like nasal lesions and collagen deposition in mice. (a) Representative H&E‐stained sections of nasal mucosa from Control and CRSwNP‐like mice (upper panels, overview; lower panels, magnified insets). (b–d) Quantification of polypoid lesion number (b; *p* = 0.0286), epithelial pathology score (c; *p* = 0.0286), and nasal mucosal epithelial thickness (d; *p* < 0.0001). (e) Sirius Red staining under bright‐field showing collagen deposition. (f) Polarized‐light microscopy of Sirius Red‐stained sections showing collagen fiber signals. (g) Collagen area fraction (*p *= 0.0438). *n* = 4 mice per group; (b, c) two‐tailed Mann–Whitney *U* test; (d, g) two‐tailed unpaired *t* test with Welch's correction; data are presented as mean ± SEM, and each dot represents one mouse; *p < 0.05, ****p < 0.0001. Scale bars = 50 μm. Source data are provided in Supporting Information S1: Tables S10–S12.

Sirius Red staining of serial sections, combined with polarized light microscopy, was used to evaluate collagen deposition. Red‐stained collagen fibers were more prominent in the subepithelial lamina propria of CRSwNP mice than in controls (Figure 6e), and polarized light revealed correspondingly stronger collagen signals (Figure 6f). Quantification confirmed a significant increase in collagen area in the CRSwNP group (*p *= 0.0438; Figure 6g), indicating that the model recapitulates both the polypoid inflammatory changes and the collagen‐deposition phenotype of CRSwNP.

### 3.8 Activation of the TGF‐β/Smad Pathway in the Nasal Mucosa of CRSwNP Mice Is Accompanied by Concomitant Enhancement of Fibroblast Activation‐Associated Collagen Deposition and Epithelial EMT

The aforementioned Sirius Red staining results suggested marked collagen deposition in the lamina propria of the nasal mucosa in CRSwNP‐like mice. As nasal fibroblasts are known to acquire an α‐SMA^+^ myofibroblast‐like phenotype while producing Type I collagen [41], we performed double immunofluorescence for α‐SMA and COL1A1. Both signals were weak in control mucosa, whereas CRSwNP mice showed subepithelial α‐SMA^+^ cell accumulation accompanied by increased COL1A1 deposition (α‐SMA, *p* = 0.0342; COL1A1, *p* = 0.0317; Figure 7a,b), in agreement with the Sirius Red findings. Given the spatial proximity between the epithelial layer and the lamina propria, we next examined epithelial E‐cadherin and nuclear p‐Smad3: E‐cadherin was reduced (*p* = 0.0295) while nuclear p‐Smad3 increased (*p *= 0.0112) in CRSwNP mice (Figure 7c,d). Single‐marker staining further showed enhanced N‐cadherin (*p *= 0.0061) and POSTN (*p *= 0.0061) signals (Figure 7e–h). Finally, colocalization analysis of α‐SMA and COL1A1 showed a tendency toward more synchronous distribution in CRSwNP mucosa, although Pearson's coefficients did not differ significantly (*p *= 0.3200; Figure 7i–k), suggesting that the two markers reflect remodeling in adjacent regions rather than strict co‐expression. Thus, TGF‐β/Smad3 activation in this model occurred concomitantly with fibroblast activation, collagen deposition, and EMT‐like epithelial changes.

**Figure 7:**
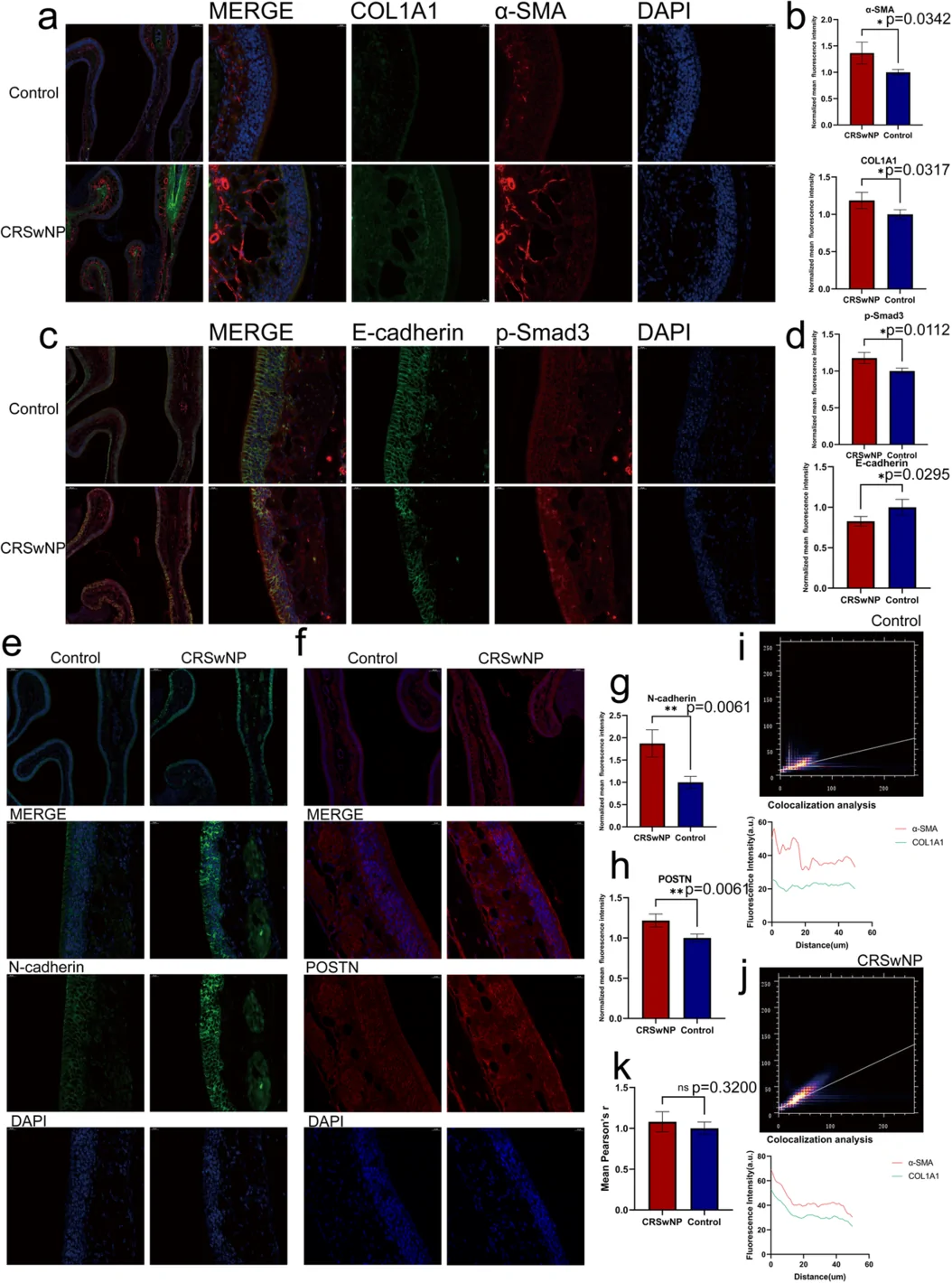
Immunofluorescence validation of fibroblast activation, epithelial EMT‐like changes, and TGF‐β/Smad3 activation in CRSwNP‐like mice. (a) Double immunofluorescence of COL1A1 (green) and α‐SMA (red). (b) Quantification of COL1A1 (*p *= 0.0317) and α‐SMA (*p *= 0.0342) in the lamina propria. (c) Double immunofluorescence of E‐cadherin (green) and p‐Smad3 (red). (d) Quantification of epithelial E‐cadherin (*p *= 0.0295) and nuclear p‐Smad3 (*p *= 0.0112). (e, f) Single‐marker immunofluorescence of N‐cadherin (e) and POSTN (f). (g, h) Quantification of N‐cadherin (g; *p* = 0.0061) and POSTN (h; *p* = 0.0061). (i, j) Colocalization analysis of α‐SMA and COL1A1 in Control (i) and CRSwNP (j) mucosa, shown as pixel‐intensity scatter plots and fluorescence‐intensity profiles. (k) Mean Pearson's correlation coefficient for α‐SMA/COL1A1 colocalization (*p *= 0.3200; ns). Nuclei were counterstained with DAPI. *n* = 4 mice per group; two‐tailed unpaired *t* test with Welch's correction; data are presented as mean ± SEM; *p < 0.05, **p < 0.01; ns, not significant. Scale bars = 25 μm. Source data are provided in Supporting Information S1: Tables S13–S15.

### 3.9 Dexamethasone Partially Reverses TGF‐β/Smad3‐Associated Remodeling at the Protein Level

Glucocorticoids, the first‐line medical therapy for CRSwNP, also antagonize TGF‐β/Smad3‐mediated profibrotic signaling [42, 43]. We therefore examined nasal mucosa from Control, CRSwNP, and dexamethasone‐treated CRSwNP (DEX) mice by western blotting. Consistent with the immunofluorescence results, CRSwNP mucosa showed increased COL1A1, POSTN, N‐cadherin and α‐SMA, reduced E‐cadherin, and an elevated p‐Smad3/Smad3 ratio (all *p* < 0.001 vs. Control), whereas total Smad3 did not differ among the three groups (all adjusted *p* > 0.9; Figure 8a–f). DEX treatment partially reversed this profile: COL1A1 (*p* = 0.010), POSTN (*p* < 0.001), N‐cadherin (*p* = 0.011) and α‐SMA (*p* < 0.001) were reduced, and E‐cadherin was restored (*p* < 0.001), relative to untreated CRSwNP mice; of these, α‐SMA and E‐cadherin returned to levels statistically indistinguishable from controls (*p* = 0.052 and *p* = 0.091, respectively), whereas COL1A1, POSTN and N‐cadherin remained above control levels (*p* = 0.043, *p* = 0.003 and *p* = 0.009, respectively). The p‐Smad3/Smad3 ratio showed only a non‐significant downward trend after DEX treatment (*p* = 0.085 vs. CRSwNP) and remained elevated relative to controls (*p* = 0.001). These protein‐level data support the presence of TGF‐β/Smad3‐associated epithelial–fibroblast remodeling in this model and indicate that it is partially reversible by corticosteroid treatment.

**Figure 8:**
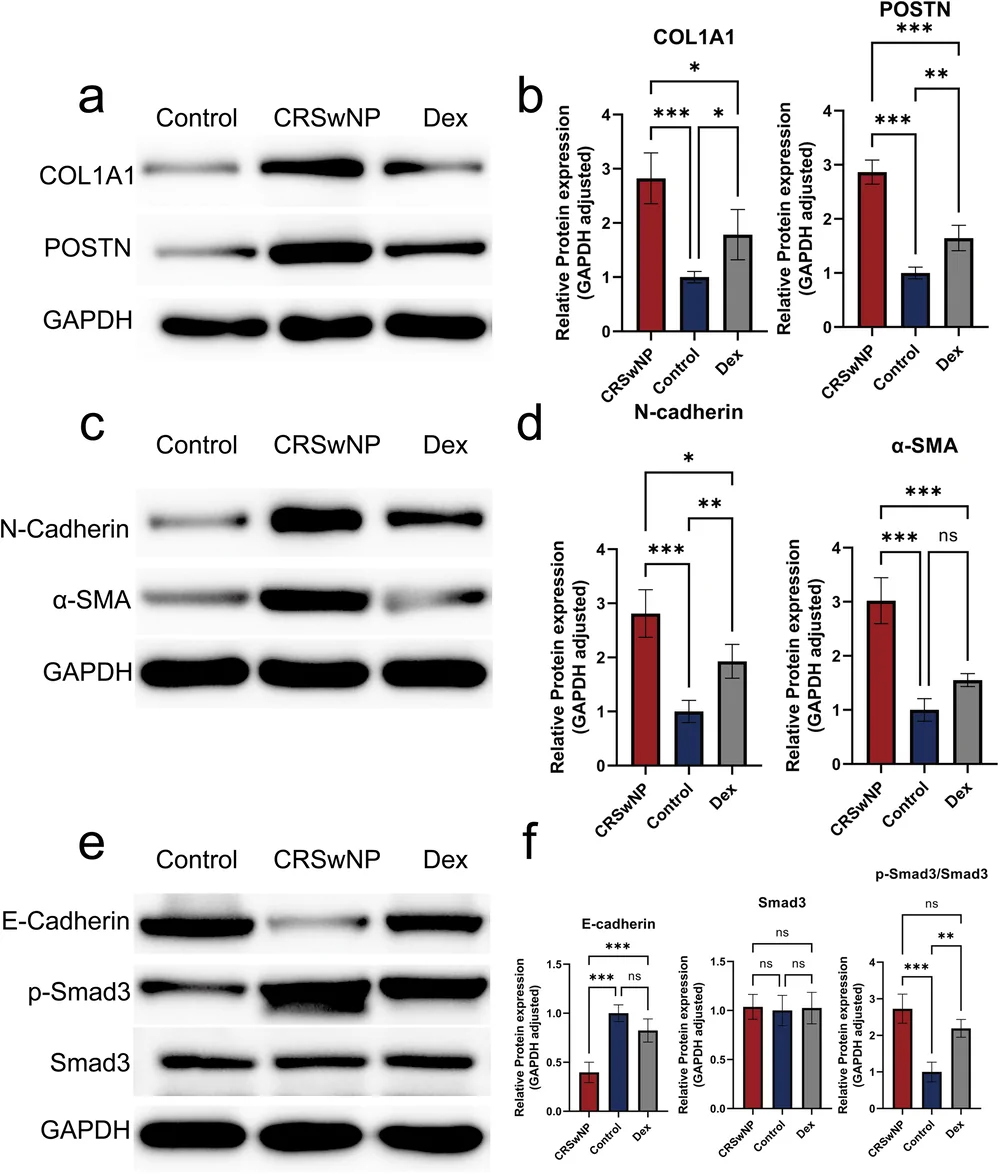
Dexamethasone partially reverses TGF‐β/Smad3‐associated remodeling at the protein level. (a, c, e) Representative immunoblots of COL1A1 and POSTN (a), N‐cadherin and α‐SMA (c), and E‐cadherin, p‐Smad3, and total Smad3 (e) in nasal mucosa from Control, CRSwNP, and dexamethasone‐treated (DEX) mice; GAPDH served as the loading control. (b, d, f) Densitometric quantification normalized to GAPDH, with p‐Smad3 additionally normalized to total Smad3 (p‐Smad3/Smad3). *n* = 4 mice per group; ordinary one‐way ANOVA followed by Tukey's multiple comparisons test; data are presented as mean ± SEM; *p < 0.05, **p < 0.01, ***p < 0.001; ns, not significant. Source data are provided in Supporting Information S1: Table S17.

## 4 Discussion

This study linked epithelial and fibroblast state changes identified by single‐cell transcriptomics with tissue‐level remodeling validated in human nasal tissue and a CRSwNP‐like mouse model. At the compositional level, epithelial cells shifted toward basal‐like states (KRT15, COL17A1) at the expense of club (SCGB1A1) and ciliated (FOXJ1) populations, with MUC5AC‐expressing secretory cells expanded, while fibroblasts moved away from homeostatic states (APOD, CFD) toward inflammatory and matrix‐associated populations (CCL2, COL1A2) (Figures 1c–f and 2a,b). CellChat further nominated COLLAGEN and TGF‐β signaling as candidate bidirectional axes between the two compartments (e.g., COL1A1–SDC4/CD44 and TGFB1–TGFBR1/TGFBR2) (Figure 4a–i); myeloid subclustering, in turn, localized TGFB1 preferentially to a GPNMB‐expressing macrophage state expanded in polyps (Supporting Information S1: Figure S10a–c). These transcriptomic findings were substantiated at the tissue and protein levels: human polyps showed collagen deposition, enrichment of α‐SMA–high activated fibroblasts, EMT‐like epithelial changes, and nuclear p‐Smad3 accumulation (Figure 5a–h); the CRSwNP‐like mouse model reproduced this pattern (Figures 6a–g and 7a–k), and dexamethasone treatment partially reversed it (Figure 8a–f).

The epithelial compartment of polyps fits a pattern of skewed injury–repair: barrier defects on one side and aberrant regeneration on the other [1, 4, 6]. Basal‐like cells marked by KRT15 and COL17A1 expanded prominently, in line with previous single‐cell observations of heightened regenerative activity in chronically inflamed nasal epithelium (Figure 2b) [16, 17]. By contrast, club (SCGB1A1) and ciliated (FOXJ1) cells contracted, a loss expected to compromise barrier integrity and mucociliary clearance, two well‐documented defects in CRS (Figure 2b) [6]. EMT‐like features extended beyond the transcriptional level: in human polyps, epithelial E‐cadherin weakened while N‐cadherin and nuclear p‐Smad3 increased (Figures 3g,h and 5c–f), echoing the partial epithelial–mesenchymal transition described in CRS epithelium [39, 44]. Because type I collagen itself can promote EMT through TGF‐β–dependent and –independent routes [45, 46, 47], including COL1A1‐mediated signaling in eosinophilic CRSwNP [15], the coexistence of stromal collagen deposition and epithelial EMT‐like changes raises the possibility of a self‐reinforcing loop between matrix accumulation and epithelial remodeling. SDC4 and CD44, both implicated in extracellular‐matrix sensing and signal transduction [36, 37], provide a molecular foothold for such matrix–epithelium communication (Figure 4e,h). These epithelial changes did not unfold in isolation.

A parallel restructuring unfolded in the fibroblast compartment (Figure 2a). This shift mirrors the conserved fibroblast‐activation axis described in cross‐tissue atlases and type I collagen lineage studies [24, 25]; pseudotime ordering likewise placed the homeostatic markers APOD, CFD and PI16 early and the collagen genes COL1A1, COL1A2 and FN1 at mid‐to‐late stages (Figure 3i,j), in keeping with gradual progression along a differentiation continuum rather than an abrupt switch. On the signaling side, the place of TGF‐β in CRS remodeling has remained incompletely resolved, with ligand abundance, receptor engagement, and downstream output not always tracking together across studies [12, 48]. Against this background, our data read pathway activity directly at the protein level: nuclear p‐Smad3 accumulated in polyp epithelium and stroma (Figures 5c,d and 7c,d), and the p‐Smad3/Smad3 ratio rose on immunoblots (Figure 8e,f), together offering one route to reconcile these discrepancies [38]. A second observation concerns the basement‐membrane zone: in control mucosa, POSTN and COL1A1 formed a compact subepithelial band, whereas in polyps this band appeared loose and discontinuous despite stronger overall fluorescence (Figure 5e,f)—a redistribution reminiscent of the basement‐membrane thickening described in refractory CRSwNP [5]. Periostin is a matricellular protein secreted downstream of IL‐4/IL‐13 signaling [49], and epithelial‐derived periostin can itself promote TGF‐β activation and collagen production [50]; it may therefore participate in basement‐membrane‐zone matrix reorganization rather than serving as a passive fibrosis marker. Together with the epithelial‐side findings, the reciprocal allocation of COLLAGEN signaling (fibroblast→epithelial) and TGF‐β signaling (epithelial→fibroblast) nominates a candidate feed‐forward circuit between the two compartments (Figure 4c–f).

Beyond these two structural compartments, immune cells are established cellular sources of TGF‐β1 in chronically inflamed upper airway tissues [10, 11, 12]. The myeloid compartment supplied a concrete cellular address for this notion: TGFB1 transcripts were preferentially detected in a GPNMB‐expressing macrophage state (SPP1, TREM2, MMP9, MSR1), a profile closely paralleling scar‐associated macrophages in fibrotic tissues [51], and this state was significantly expanded in polyps (Supporting Information S1: Figure S10a–c). Within this landscape, immune‐cell‐derived TGF‐β1 stands as one contributing component: epithelial and fibroblast subpopulations occupied the dominant sender positions in the TGF‐β and COLLAGEN networks (Figure 4a–c) and are the cells that physically execute tissue remodeling, so our mechanistic focus remains on this axis. Eosinophils, a classic TGF‐β1 source in nasal polyps [10], were too sparsely captured in either dataset to support subcluster‐level analysis (see Limitations). How TGF‐β1 production is partitioned between immune and structural compartments, and how this partition shifts across inflammatory endotypes, awaits spatially resolved and functional interrogation.

To test whether these transcriptional inferences hold at the tissue level, we turned to human polyp specimens and a CRSwNP‐like mouse model. Immunofluorescence and histological staining of human polyps confirmed the core predictions, including collagen accumulation, enrichment of α‐SMA–high activated fibroblasts, weakened epithelial E‐cadherin, induction of N‐cadherin and POSTN, and nuclear accumulation of p‐Smad3 (Figure 5a–h). Rather than the ligand itself, nuclear p‐Smad3 was used to gauge pathway activation: TGF‐β1 is secreted in latent form and tissue abundance poorly reflects signaling flux, whereas Smad3 phosphorylation and nuclear translocation mark direct receptor‐mediated output [38, 48]. In vivo, the OVA/SEB‐induced CRSwNP‐like model recapitulated both the histological hallmarks [35] (polypoid lesions, epithelial thickening, and subepithelial collagen deposition; Figure 6a–g) and the molecular signature seen in human polyps (Figure 7a–k). Glucocorticoids remain the first‐line anti‐inflammatory therapy for CRSwNP [2, 34, 43]. This choice has a mechanistic footing: the glucocorticoid receptor directly targets the transcriptional‐activation function of Smad3 [42], and pathway‐directed interventions have previously alleviated TGF‐β1‐induced nasal mucosal remodeling [14, 41]. Dexamethasone partially reversed the remodeling phenotype: α‐SMA and E‐cadherin returned essentially to control levels, COL1A1, POSTN, and N‐cadherin declined but remained above control, and the p‐Smad3/Smad3 ratio showed only a downward trend (*p* = 0.085) (Figure 8a–f). Such partial reversal is itself informative. Dexamethasone is a broad‐spectrum anti‐inflammatory agent rather than a pathway‐specific inhibitor; the incomplete normalization of Smad3 signaling suggests that anti‐inflammatory treatment alone may not suffice to reverse established remodeling and that pathway‐targeted anti‐remodeling strategies deserve exploration.

These convergent lines of evidence should nonetheless be weighed against several limitations. First, the CellChat and trajectory analyses are computational inferences from transcriptomic data: they nominate candidate axes rather than demonstrate physical ligand–receptor engagement or causality, and will require validation by spatially resolved transcriptomics and perturbation‐based assays [33]. Second, the two public cohorts differ in ethnic and endotypic background: GSE235711 derives from a North American cohort skewed toward Type 2 inflammation [16], whereas GSE276503 originates from a Chinese cohort characterized by Interleukin‐1β (IL‐1β)–related myeloid inflammation [13], and such inflammatory heterogeneity is well documented in CRS [17]. Nor was the analysis stratified by sinonasal site; tissue origin (turbinate vs. septal mucosa vs. polyp), epithelial type (respiratory vs. olfactory), and clinical variables (sampling technique, storage time, and disease course) could not be fully harmonized. Nevertheless, the reproducible detection of the epithelial–fibroblast TGF‐β/Smad axis and its collagen‐deposition program across two cohorts differing in ethnicity, endotype, sequencing platform, and sampling site argues that this axis represents a shared remodeling feature rather than a cohort‐specific artifact. Eosinophils in particular were under‐captured: few reside in control turbinates, the two cohorts include non‐eosinophilic endotypes, and granulocytes are fragile and RNA‐poor in droplet‐based scRNA‐seq, leaving fewer than 1000 cells after quality control, below the threshold for reliable subcluster‐level analysis. Third, the OVA/SEB model carries a Type 2–skewed, eosinophilic background that does not fully correspond to the myeloid inflammation emphasized in GSE276503, and the inherent constraints of this model have been systematically documented [35, 40]. Furthermore, causal relationships were not tested: neither COL1A1 knockdown, TGF‐β/Smad pathway blockade, nor epithelial–fibroblast co‐culture was performed.

This study was not intended to establish a precise stratified model for any single inflammatory endotype of CRSwNP, but rather takes tissue remodeling, a common pathological manifestation of CRSwNP, as its entry point. By integrating human single‐cell transcriptomics with histological and protein‐level validation in both human tissue and a CRSwNP‐like animal model, it bridges cellular‐state alterations with tissue‐level remodeling pathology. Whereas previous single‐cell studies of CRSwNP have centered on immune inflammation and immune–epithelial interactions [13, 16, 17], the present work extends the perspective to a pathological pattern jointly driven by aberrant repair and matrix remodeling. Such a shift may help explain the refractoriness, recurrence, and structural persistence of nasal polyps. The epithelial–fibroblast interactions and microenvironmental remodeling features delineated here offer potential directions for anti‐remodeling therapy and an experimental basis for pathway‐blockade and interaction‐validation studies.

## 5 Conclusion

Integrating single‐cell transcriptomics of two independent human cohorts with protein‐level validation in human polyp tissue and a CRSwNP‐like mouse model, this study characterizes a TGF‐β/Smad3‐associated epithelial–fibroblast remodeling program underlying collagen deposition in CRSwNP. Within this program, basal‐shifted, EMT‐like epithelial changes accompany fibroblast activation and collagen accumulation, with nuclear p‐Smad3 activation across both compartments and partial reversal by dexamethasone in vivo. Cell‐communication analysis points to bidirectional COLLAGEN and TGF‐β signaling as candidate intercellular axes, and identifies a GPNMB‐expressing macrophage state as an additional cellular source of TGFB1. The epithelial–fibroblast TGF‐β/Smad3 axis thus emerges as a cross‐cohort feature of polyp remodeling and a tractable direction for anti‐remodeling intervention, pending spatially resolved and functional validation.

## Author Contributions

**Shilong Liu:** conceptualization (equal), methodology (lead), software (lead), formal analysis (lead), investigation (lead), data curation (lead), visualization (lead), writing – original draft (lead), writing – review and editing (equal). **Peizheng Xie:** investigation (supporting), data curation (equal), writing – review and editing (equal). **Hanwei Deng:** investigation (supporting), data curation (equal), writing – review and editing (equal). **Zhenhua Zhu:** conceptualization (equal); methodology (supporting), supervision (lead), project administration (lead), funding acquisition (lead), writing – review and editing (lead). Peizheng Xie and Hanwei Deng contributed equally to this work. All authors reviewed and approved the final version of the manuscript.

## Ethics Statement

The animal study protocol was approved by the Experimental Animal Ethics Committee of the First Affiliated Hospital of Hunan University of Chinese Medicine (protocol code 202508201; date of approval: September 1, 2025). The clinical study was conducted in accordance with the Declaration of Helsinki and was approved by the Ethics Review Committee of the First Affiliated Hospital of Hunan University of Chinese Medicine (protocol code HN‐LL‐YJSLW‐2026‐25; date of approval: January 1, 2026). The requirement for written informed consent was waived by the committee as part of the approved protocol, given that the study used only residual surgical specimens and involved no identifiable personal information.

## Conflicts of Interest

The authors declare no conflicts of interest.

## Supporting information


Supporting File

## Data Availability

The single‐cell RNA sequencing datasets analyzed in this study are publicly available in the Gene Expression Omnibus database under accession numbers GSE235711 and GSE276503. Additional data generated or analyzed during this study are available from the corresponding author upon reasonable request.
