# Supplementary material for: TGF‐β/Smad‐Associated Epithelial–Fibroblast Remodeling Underlies Collagen Deposition in Chronic Rhinosinusitis With Nasal Polyps
Source: Immun Inflamm Dis. 2026 Oct 6;14(10):e70532. doi: 10.1002/iid3.70532 (PMC13640533; doi:10.1002/iid3.70532)
Supplement: Supplementary file 1 [file IID3-14-e70532-s001.zip › Supplementary material 9.30 ver/Figure/S1.before_QC.pdf]

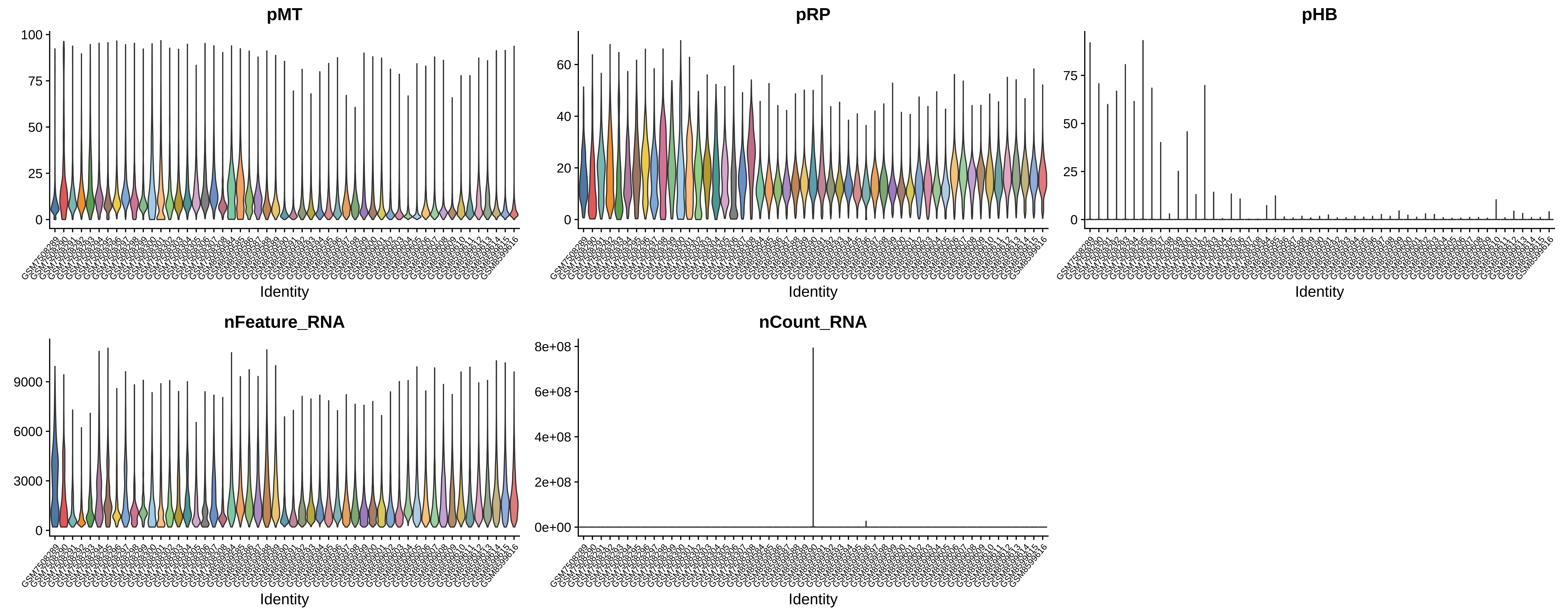

pMT
pRP
pHB
nFeature_RNA
nCount_RNA
Identity
100
75
50
25
0
60
40
20
9000
6000
3000
8e+08
6e+08
4e+08
2e+08
0e+00
GSM7508289
GSM8599616
