# Supplementary material for: TGF‐β/Smad‐Associated Epithelial–Fibroblast Remodeling Underlies Collagen Deposition in Chronic Rhinosinusitis With Nasal Polyps
Source: Immun Inflamm Dis. 2026 Oct 6;14(10):e70532. doi: 10.1002/iid3.70532 (PMC13640533; doi:10.1002/iid3.70532)
Supplement: Supplementary file 1 [file IID3-14-e70532-s001.zip › Supplementary material 9.30 ver/Figure/S10. Macro.pdf]

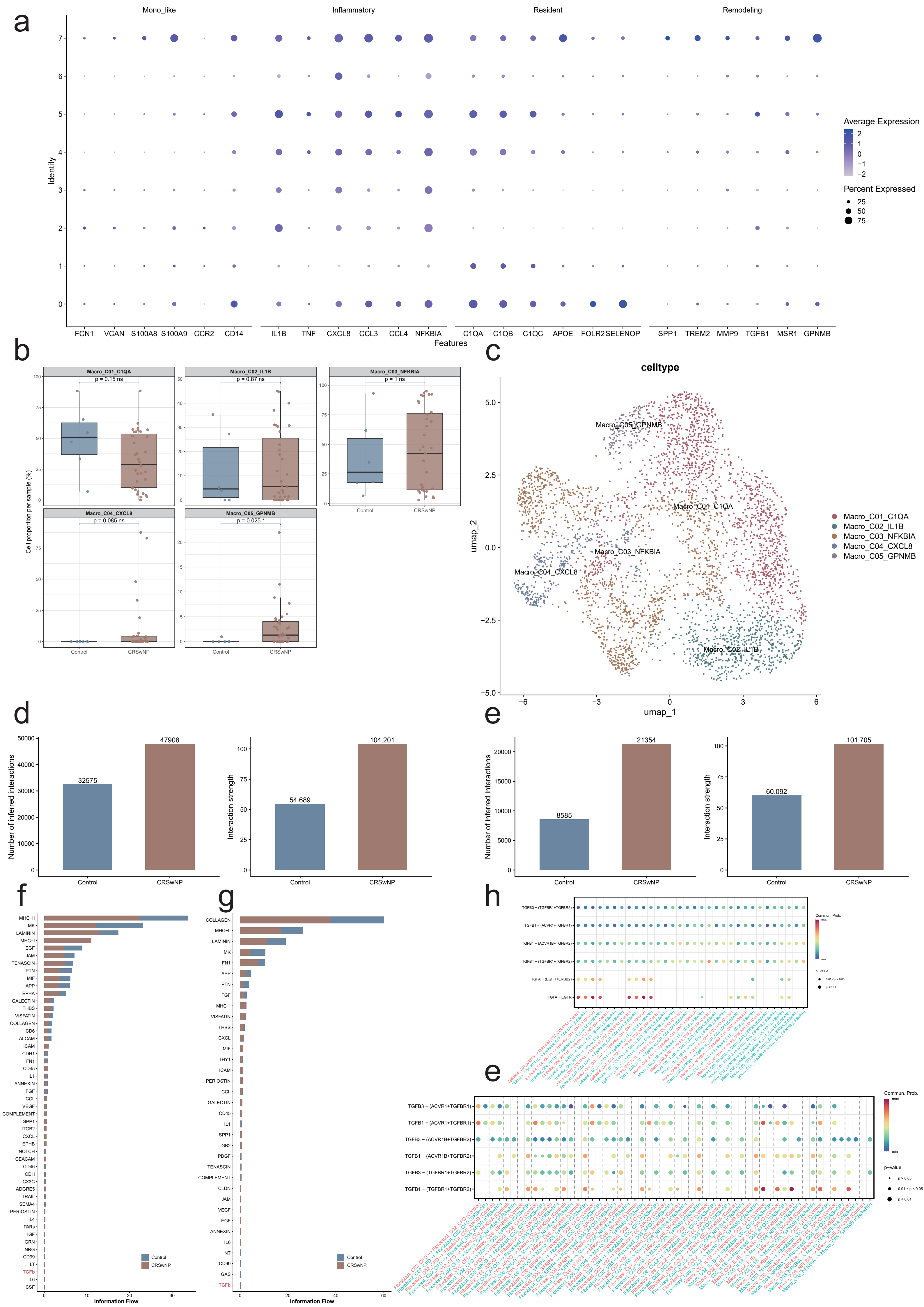
a
Mono_like
Inflammatory
Resident
Remodeling
Identity
7
6
5
4
3
2
1
0
FCN1
VCAN
S100A8
S100A9
CCR2
CD14
IL1B
TNF
CXCL8
CCL3
CCL4
NFKBIA
C1QA
C1QB
C1QC
APOE
FOLR2
SELENOP
SPP1
TREM2
MMP9
TGFB1
MSR1
GPNMB
Features
Average Expression
2
1
0
−1
−2
Percent Expressed
25
50
75
b
Macro_C01_C1QA
p = 0.15 ns
Macro_C02_IL1B
p = 0.87 ns
Macro_C03_NFKBIA
p = 1 ns
Macro_C04_CXCL8
p = 0.085 ns
Macro_C05_GPNMB
p = 0.025 *
Cell proportion per sample (%)
Control
CRSwNP
c
celltype
Macro_C05_GPNMB
Macro_C01_C1QA
Macro_C03_NFKBIA
Macro_C04_CXCL8
Macro_C02_IL1B
umap_2
umap_1
Macro_C01_C1QA
Macro_C02_IL1B
Macro_C03_NFKBIA
Macro_C04_CXCL8
Macro_C05_GPNMB
d
Number of inferred interactions
32575
47908
Control
CRSwNP
Interaction strength
54.689
104.201
Control
CRSwNP
e
Number of inferred interactions
8585
21354
Control
CRSwNP
Interaction strength
60.092
101.705
Control
CRSwNP
f
MHC−II
MK
LAMININ
MHC−I
EGF
JAM
TENASCIN
PTN
MIF
APP
EPHA
GALECTIN
THBS
VISFATIN
COLLAGEN
CD6
ALCAM
ICAM
CDH1
FN1
CD45
IL1
ANNEXIN
FGF
CCL
VEGF
COMPLEMENT
SPP1
ITGB2
CXCL
EPHB
NOTCH
CEACAM
CD46
CDH
CX3C
ADGRE5
TRAIL
SEMA4
PERIOSTIN
IL4
PARs
IGF
GRN
NRG
CD99
LT
TGFb
IL6
CSF
Control
CRSwNP
Information Flow
g
COLLAGEN
MHC−II
LAMININ
MK
FN1
APP
PTN
FGF
MHC−I
VISFATIN
THBS
CXCL
MIF
THY1
ICAM
PERIOSTIN
CCL
GALECTIN
CD45
IL1
SPP1
ITGB2
PDGF
TENASCIN
COMPLEMENT
CLDN
JAM
VEGF
EGF
ANNEXIN
IL6
NT
CD99
GAS
TGFb
Control
CRSwNP
Information Flow
h
TGFB3 − (TGFBR1+TGFBR2)
TGFB1 − (ACVR1+TGFBR1)
TGFB1 − (ACVR1B+TGFBR2)
TGFB1 − (TGFBR1+TGFBR2)
TGFA − (EGFR+ERBB2)
TGFA − EGFR
Commun. Prob.
p-value
e
TGFB3 − (ACVR1+TGFBR1)
TGFB1 − (ACVR1+TGFBR1)
TGFB3 − (ACVR1B+TGFBR2)
TGFB1 − (ACVR1B+TGFBR2)
TGFB3 − (TGFBR1+TGFBR2)
TGFB1 − (TGFBR1+TGFBR2)
Commun. Prob.
max
min
p−value
p > 0.05
0.01 < p < 0.05
p < 0.01
