# Supplementary material for: TGF‐β/Smad‐Associated Epithelial–Fibroblast Remodeling Underlies Collagen Deposition in Chronic Rhinosinusitis With Nasal Polyps
Source: Immun Inflamm Dis. 2026 Oct 6;14(10):e70532. doi: 10.1002/iid3.70532 (PMC13640533; doi:10.1002/iid3.70532)
Supplement: Supplementary file 1 [file IID3-14-e70532-s001.zip › Supplementary material 9.30 ver/Figure/S2.after_QC.pdf]

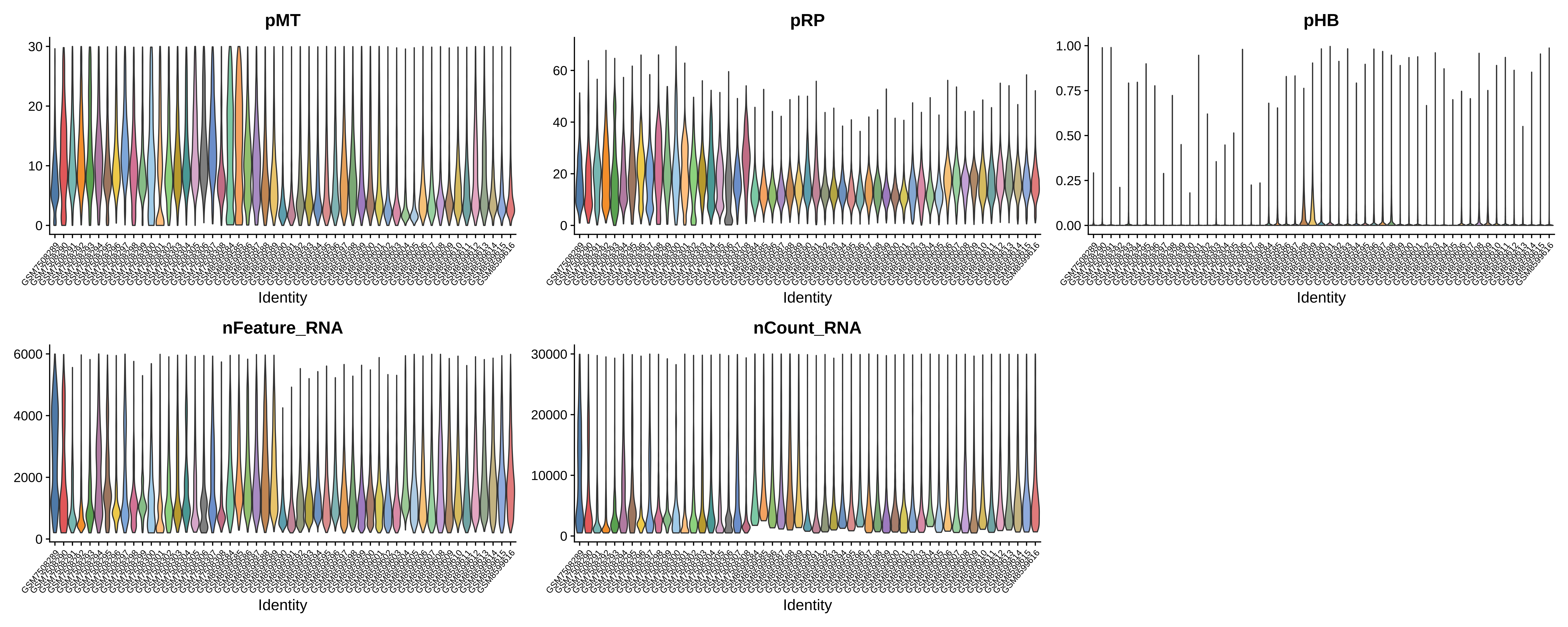
pMT
pRP
pHB
nFeature_RNA
nCount_RNA
Identity
Identity
Identity
Identity
Identity
0
10
20
30
0
20
40
60
0.00
0.25
0.50
0.75
1.00
0
2000
4000
6000
0
10000
20000
30000
