# Supplementary material for: TGF‐β/Smad‐Associated Epithelial–Fibroblast Remodeling Underlies Collagen Deposition in Chronic Rhinosinusitis With Nasal Polyps
Source: Immun Inflamm Dis. 2026 Oct 6;14(10):e70532. doi: 10.1002/iid3.70532 (PMC13640533; doi:10.1002/iid3.70532)
Supplement: Supplementary file 1 [file IID3-14-e70532-s001.zip › Supplementary material 9.30 ver/Figure/S4.rna_contamination.pdf]

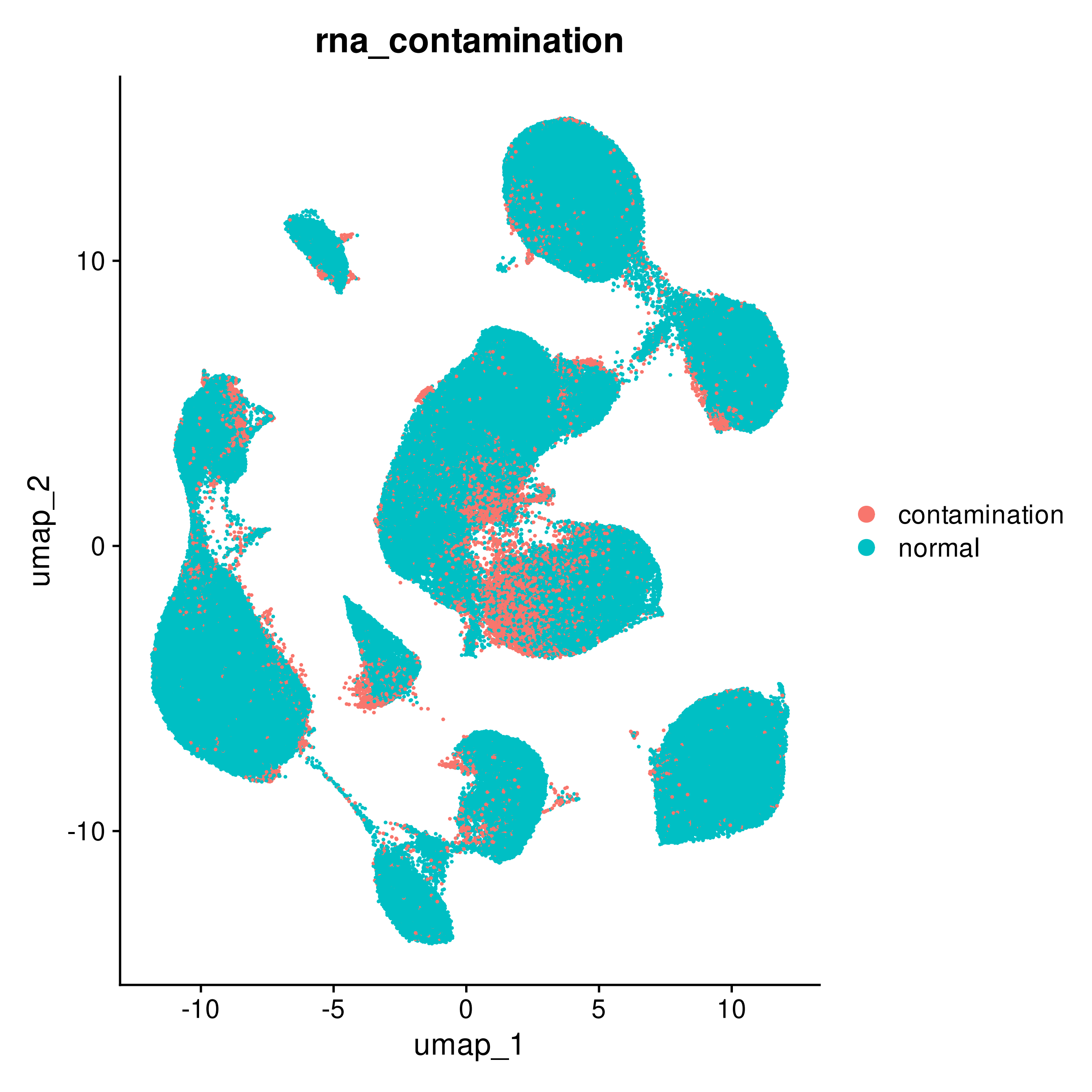
rna_contamination
umap_2
umap_1
10
0
-10
-10
-5
0
5
10
contamination
normal
