# Supplementary material for: TGF‐β/Smad‐Associated Epithelial–Fibroblast Remodeling Underlies Collagen Deposition in Chronic Rhinosinusitis With Nasal Polyps
Source: Immun Inflamm Dis. 2026 Oct 6;14(10):e70532. doi: 10.1002/iid3.70532 (PMC13640533; doi:10.1002/iid3.70532)
Supplement: Supplementary file 1 [file IID3-14-e70532-s001.zip › Supplementary material 9.30 ver/Figure/S6.CytoTRACE2_Relative_VlnPlot_Epithelial.pdf]

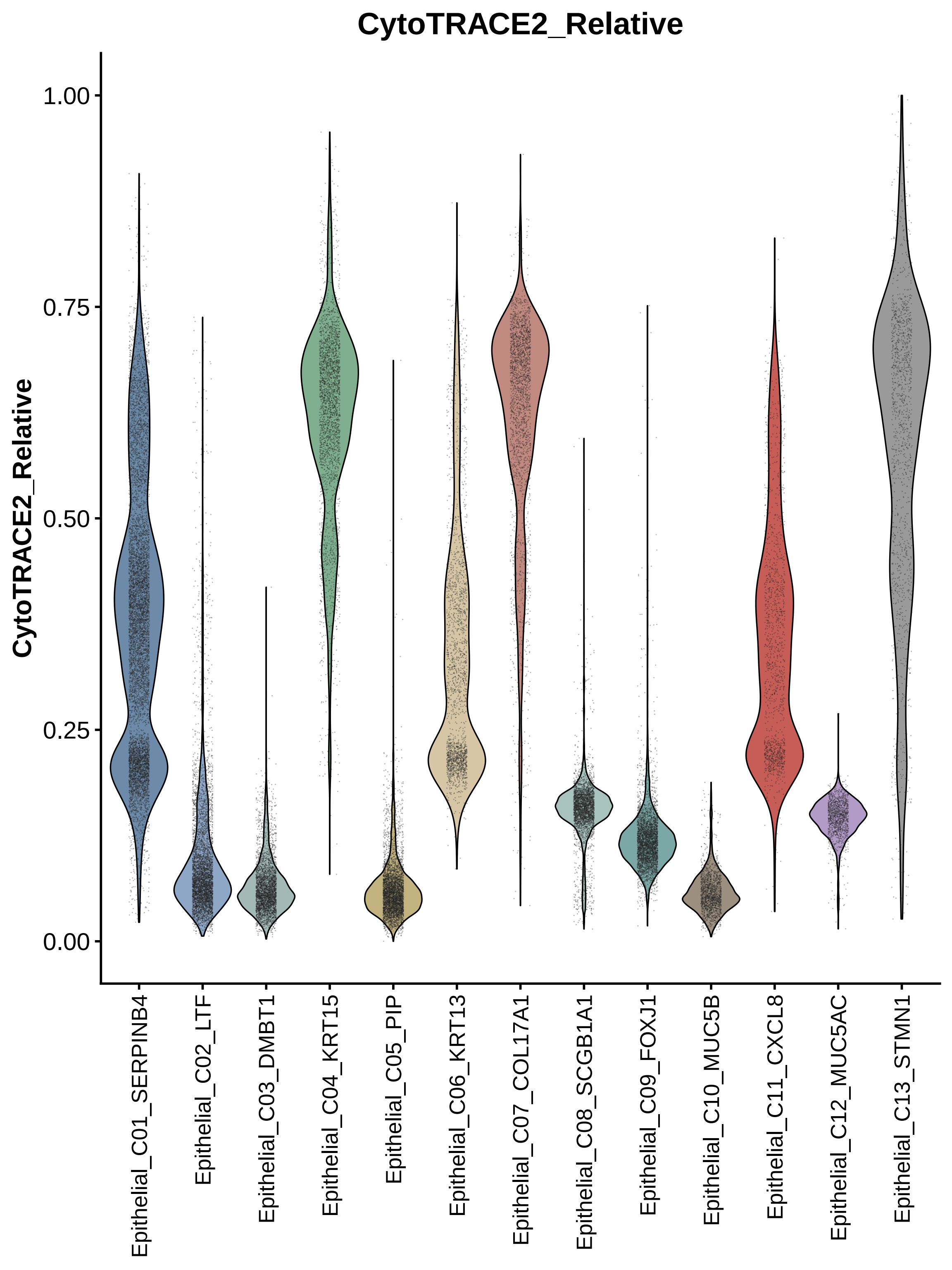
CytoTRACE2_Relative
CytoTRACE2_Relative
1.00
0.75
0.50
0.25
0.00
Epithelial_C01_SERPINB4
Epithelial_C02_LTF
Epithelial_C03_DMBT1
Epithelial_C04_KRT15
Epithelial_C05_PIP
Epithelial_C06_KRT13
Epithelial_C07_COL17A1
Epithelial_C08_SCGB1A1
Epithelial_C09_FOXJ1
Epithelial_C10_MUC5B
Epithelial_C11_CXCL8
Epithelial_C12_MUC5AC
Epithelial_C13_STMN1
