# Supplementary material for: TGF‐β/Smad‐Associated Epithelial–Fibroblast Remodeling Underlies Collagen Deposition in Chronic Rhinosinusitis With Nasal Polyps
Source: Immun Inflamm Dis. 2026 Oct 6;14(10):e70532. doi: 10.1002/iid3.70532 (PMC13640533; doi:10.1002/iid3.70532)
Supplement: Supplementary file 1 [file IID3-14-e70532-s001.zip › Supplementary material 9.30 ver/Figure/S8.CytoTRACE2_Relative_VlnPlot_fibroblast.pdf]

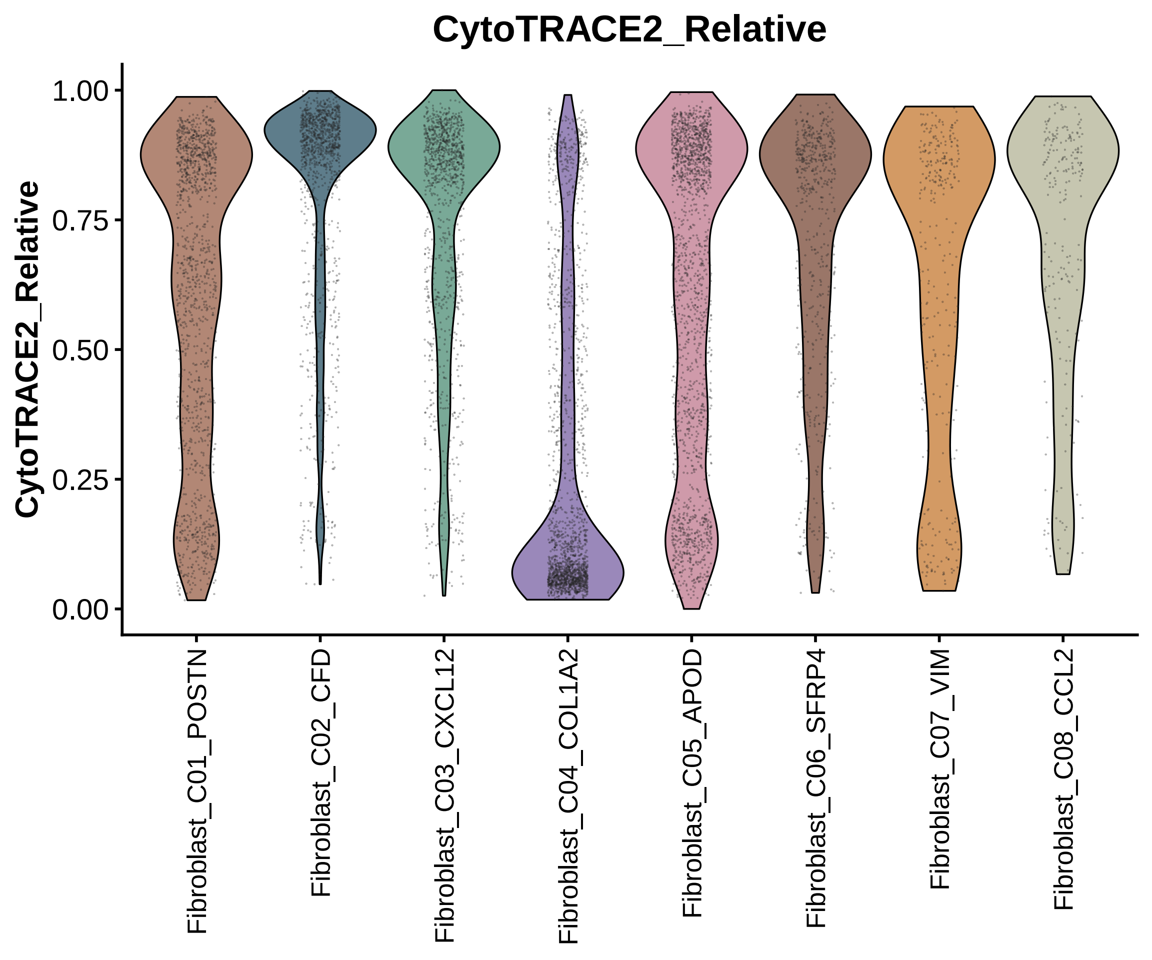

CytoTRACE2_Relative
CytoTRACE2_Relative
1.00
0.75
0.50
0.25
0.00
Fibroblast_C01_POSTN
Fibroblast_C02_CFD
Fibroblast_C03_CXCL12
Fibroblast_C04_COL1A2
Fibroblast_C05_APOD
Fibroblast_C06_SFRP4
Fibroblast_C07_VIM
Fibroblast_C08_CCL2
