# Supplementary material for: TGF‐β/Smad‐Associated Epithelial–Fibroblast Remodeling Underlies Collagen Deposition in Chronic Rhinosinusitis With Nasal Polyps
Source: Immun Inflamm Dis. 2026 Oct 6;14(10):e70532. doi: 10.1002/iid3.70532 (PMC13640533; doi:10.1002/iid3.70532)
Supplement: Supplementary file 1 [file IID3-14-e70532-s001.zip › Supplementary material 9.30 ver/Figure/S9.heatmap_in_out_compare.pdf]

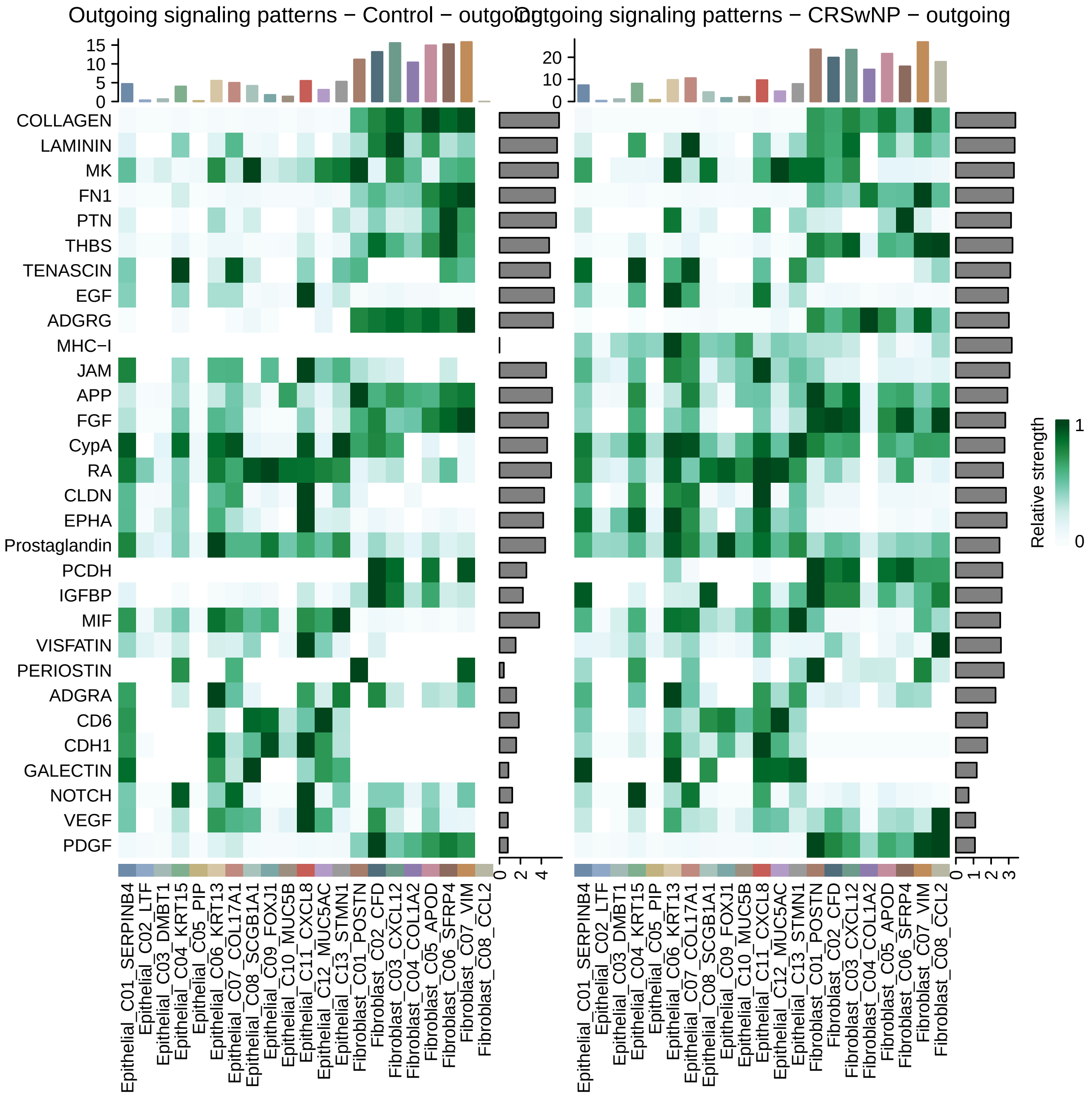
Outgoing signaling patterns − Control − outgoing
Outgoing signaling patterns − CRSwNP − outgoing
COLLAGEN
LAMININ
MK
FN1
PTN
THBS
TENASCIN
EGF
ADGRG
MHC−I
JAM
APP
FGF
CypA
RA
CLDN
EPHA
Prostaglandin
PCDH
IGFBP
MIF
VISFATIN
PERIOSTIN
ADGRA
CD6
CDH1
GALECTIN
NOTCH
VEGF
PDGF
Relative strength
Epithelial_C01_SERPINB4
Epithelial_C02_LTF
Epithelial_C03_DMBT1
Epithelial_C04_KRT15
Epithelial_C05_PIP
Epithelial_C06_KRT13
Epithelial_C07_COL17A1
Epithelial_C08_SCGB1A1
Epithelial_C09_FOXJ1
Epithelial_C10_MUC5B
Epithelial_C11_CXCL8
Epithelial_C12_MUC5AC
Epithelial_C13_STMN1
Fibroblast_C01_POSTN
Fibroblast_C02_CFD
Fibroblast_C03_CXCL12
Fibroblast_C04_COL1A2
Fibroblast_C05_APOD
Fibroblast_C06_SFRP4
Fibroblast_C07_VIM
Fibroblast_C08_CCL2

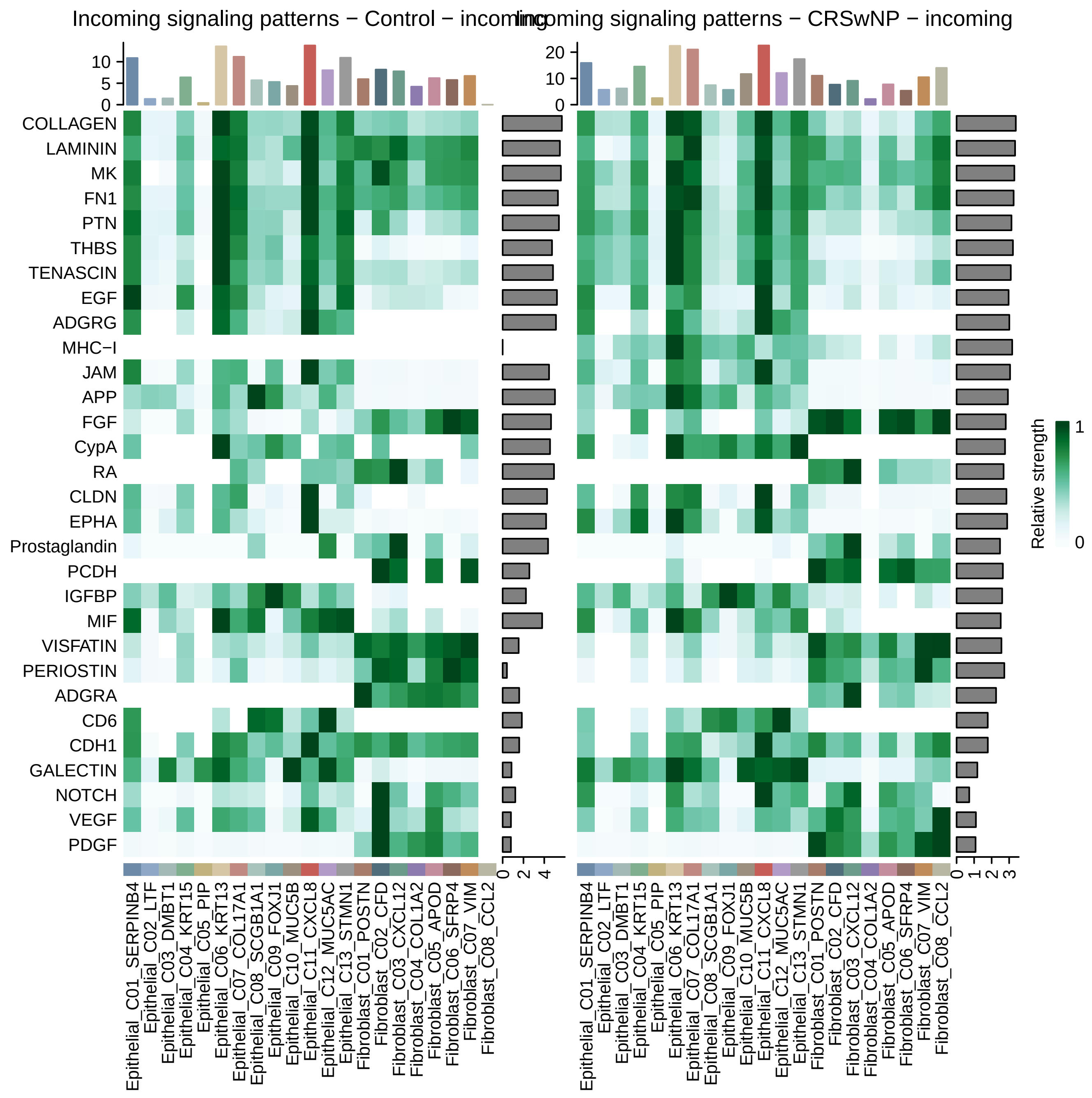

Incoming signaling patterns − Control − incoming
Incoming signaling patterns − CRSwNP − incoming
COLLAGEN
LAMININ
MK
FN1
PTN
THBS
TENASCIN
EGF
ADGRG
MHC−I
JAM
APP
FGF
CypA
RA
CLDN
EPHA
Prostaglandin
PCDH
IGFBP
MIF
VISFATIN
PERIOSTIN
ADGRA
CD6
CDH1
GALECTIN
NOTCH
VEGF
PDGF
Epithelial_C01_SERPINB4
Epithelial_C02_LTF
Epithelial_C03_DMBT1
Epithelial_C04_KRT15
Epithelial_C05_PIP
Epithelial_C06_KRT13
Epithelial_C07_COL17A1
Epithelial_C08_SCGB1A1
Epithelial_C09_FOXJ1
Epithelial_C10_MUC5B
Epithelial_C11_CXCL8
Epithelial_C12_MUC5AC
Epithelial_C13_STMN1
Fibroblast_C01_POSTN
Fibroblast_C02_CFD
Fibroblast_C03_CXCL12
Fibroblast_C04_COL1A2
Fibroblast_C05_APOD
Fibroblast_C06_SFRP4
Fibroblast_C07_VIM
Fibroblast_C08_CCL2
Relative strength
